# Optimizing Submillimeter 3D Modeling with Auxiliary Lighting and Artificial Textures: An SfM-Based Approach

**DOI:** 10.12688/f1000research.157676.2

**Published:** 2026-02-10

**Authors:** Francisco Roza de Moraes, Irineu da Silva

**Affiliations:** 1 Department of Transportation Engineering, University of Sao Paulo Sao Carlos School of Engineering, São Carlos, State of São Paulo, 13563-120, Brazil

**Keywords:** Structure from Motion, Auxiliary Lighting, Surface Artificial Texture, Storage Format, Scale Bars, Cloud Points, Root Mean Square Error

## Abstract

**Background:**

This study examines the influence of auxiliary lighting configurations and artificial surface textures on the quality of 3D models generated using Structure from Motion (SfM) in an indoor laboratory setting.

**Method:**

Experiments were conducted by capturing images of concrete, metal, and wooden specimens at a one-meter distance. Various lighting setups, including vertical and adjacent auxiliary lighting models, were tested to determine their impact on model accuracy. In addition, complex artificial textures, such as checkerboard patterns, were applied to the specimens to assess their effect on 3D model precision.

**Results:**

Our results demonstrate that optimal lighting and artificial textures significantly enhance the accuracy of 3D models, especially for materials with uniform textures, such as painted metal. For materials with more varied textures, such as concrete and wood, improvements were notable but less pronounced. The combination of auxiliary lighting and artificial textures improved model quality by approximately 40% for high-texture materials and up to 60% for uniform-texture materials. Furthermore, the study highlights the role of image file formats in the SfM process. While RAW images stored in TIFF format offered a slight advantage over lossless JPEG in terms of model accuracy, the difference may not be substantial enough to justify the larger file size in situations where submillimeter precision is not required.

**Conclusions:**

Overall, our findings emphasize the importance of tailored lighting and texturing strategies for achieving high-precision 3D models in SfM applications. These results are particularly relevant for structural testing and other applications that demand high-fidelity 3D reconstructions, providing a foundation for more accurate and reliable models.

## Introduction

The Structure from Motion (SfM) technique, developed from significant advances in Computer Vision, has become a widely adopted method for three-dimensional modeling from sets of two-dimensional images. The combination of low-cost equipment with user-friendly computational applications has contributed to the widespread popularity of this 3D modeling technique across various fields. In the academic context, for instance, the versatility of the SfM technique has enabled the production of high-quality models, facilitating detailed research, enhancing the documentation of historical heritage, and promoting the precise analysis of architectural and natural structures, as well as laboratory-scale engineering applications requiring high spatial resolution.

In Creus et al. (2021), it was noted the development of high-quality 3D products is related to factors such as the precision of control points, the use of camera calibration techniques, and the quality of the information about the set of images used. The latter factor is often partially neglected by users who, when capturing images in long-distance environments, tend to focus solely on the configuration of the photographic equipment without adequately assessing the scene’s characteristics and the object to be imaged. Recent studies have further highlighted that acquisition conditions, particularly in close-range scenarios, can exert an influence on reconstruction quality comparable to that of processing parameters and software choice ( James et al. 2017; Nielsen et al. 2023).

However, in short-distance environments with varying lighting conditions and objects with low levels of texture, these characteristics are crucial for obtaining high-quality images, which are fundamental for achieving accurate modeling. This is because the SfM technique relies on the correlation of points between images to estimate three-dimensional information about the scene. In indoor laboratory environments, even subtle variations in illumination and surface reflectance may significantly affect feature detection and matching stability.

Therefore, to efficiently execute the 3D modeling process, especially for indoor environments with short-distance captures, the analyzed environment must have consistent lighting without variations in brightness or shadowed regions. The captured object must also have a sufficiently detailed surface texture to generate accurate correlation points. Uniform lighting conditions have been shown to improve the robustness of camera calibration and image matching by reducing radiometric inconsistencies across image sets ( Dauvin et al. 2018; Nielsen et al. 2023).

Furthermore, the image storage format is another factor that is sometimes overlooked in SfM works but can significantly influence the quality of the modeling. Depending on the settings used by users, this can lead to either a loss of scene information or excessive consumption of storage space ( Reznicek et al. 2016). While compressed formats such as JPEG are widely adopted due to their reduced file size, RAW-derived formats stored as TIFF preserve a larger dynamic range and radiometric fidelity, which may be advantageous in applications targeting submillimeter accuracy ( Verma and Bourke 2019).

In the literature, there are few 3D modeling studies focused on the use of the SfM technique in indoor and short-range acquisition environments that comprehensively address these factors. Therefore, this study aims to investigate the effect of different auxiliary lighting configurations combined with the application of artificial textures on specimens made of varied materials (concrete, metal, and wood), simulating the use of the technique in laboratory testing of structural beams requiring millimeter accuracy or less. This gap is particularly relevant given recent benchmarking studies indicating that capture strategy and environmental control strongly affect close-range SfM performance ( Nikolov and Madsen 2016; Kingsland 2020).

These materials were selected due to their widespread use in structural beam testing and the distinct characteristics of their surface textures, which facilitated an accurate evaluation of the lighting configurations and artificial textures applied. Additionally, captures were conducted at an approximate distance of one meter to replicate the laboratory environment and adhere to the safety restrictions inherent to structural test protocols. Such constraints are representative of real-world laboratory conditions and directly influence acquisition strategy design.

The experiments underscored the benefits of using well-placed auxiliary lighting and the improvements provided by employing semi-closed patterns of artificial textures, which increased the accuracy of the generated models. In terms of storage formats, the use of RAW format (TIFF) showed a slight advantage over the lossless JPG format. Therefore, the results show that this technique, with careful consideration of the factors addressed in this study, applies to the modeling of structural beam tests and provides empirical guidance for optimizing acquisition-stage protocols in close-range SfM applications.

### Efficient capture techniques for SfM

**Figure 1.**
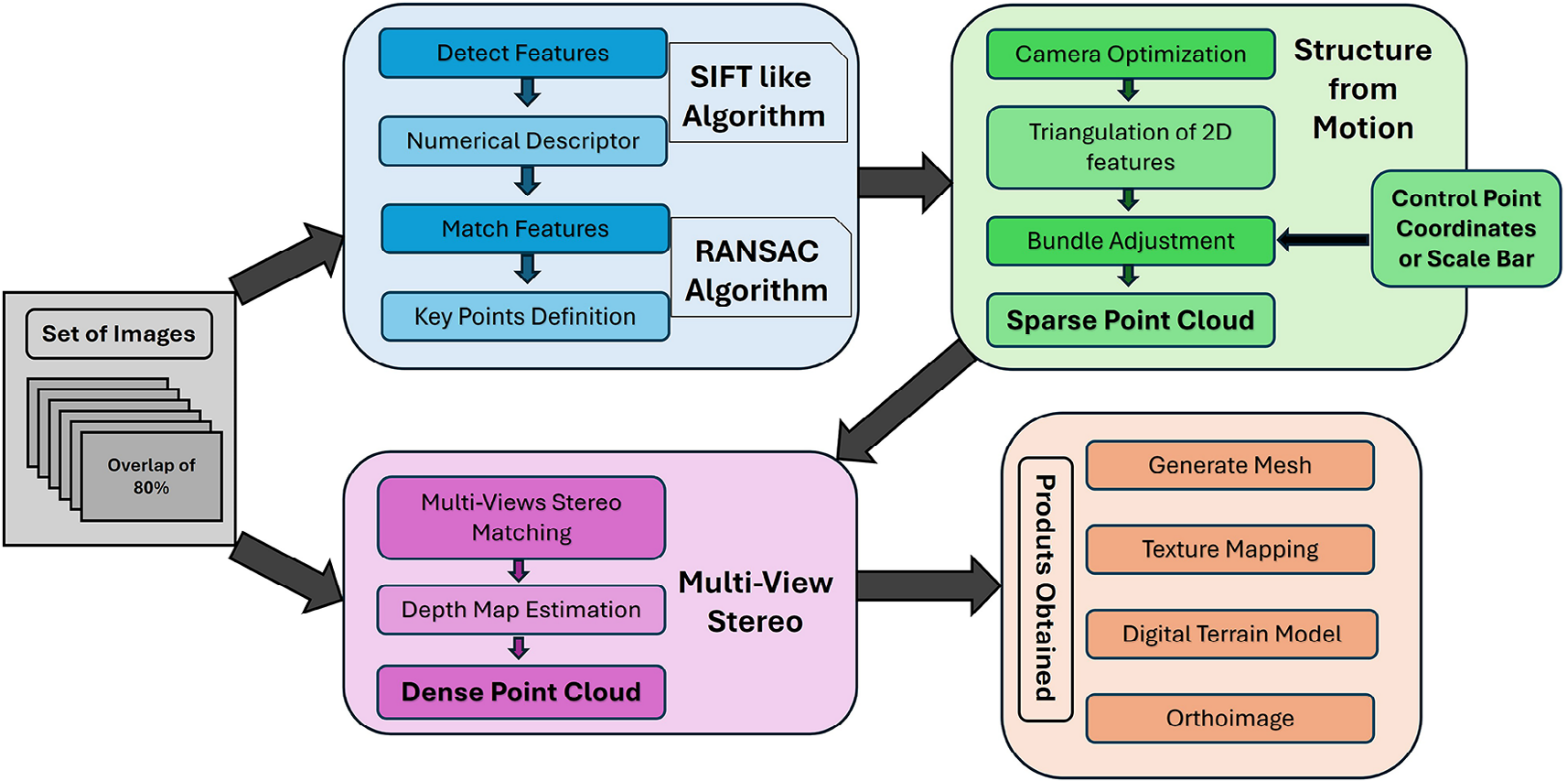
Workflow of the Structure from Motion Multi-Views Stereo process for a set of images to produce 3D modeling.

The Structure-from-Motion (SfM) technique for 3D modeling has been extensively utilized across numerous application domains, primarily due to its high degree of automation enabled by advanced computer vision methods, which contribute to the development of efficient workflows. Figure 1 illustrates a summarized overview of the standard SfM workflow.

The process begins with the acquisition of a series of images from multiple viewpoints, ensuring significant overlap between consecutive captures. Feature detection is performed on each image, followed by the matching of these features across the entire image set. The SfM phase then employs these redundant feature points to execute Bundle Adjustment, which, through the incorporation of control points or scale bars, generates a sparse point cloud that approximates the geometry of the object or scene ( James et al. 2017). This sparse representation is further refined during the Multi-View Stereo stage, where additional feature detection and matching techniques are applied to produce a dense point cloud. The result is a high-fidelity three-dimensional reconstruction of the captured object or scene ( Luhmann et al. 2020). In this workflow, the stability of feature matching across images is a key prerequisite for reliable camera pose estimation and, consequently, for the accuracy of the reconstructed geometry.

As demonstrated in the workflow, the set of input images is integral to the feature detection stages and is repeatedly utilized during the Multi-View Stereo (MVS) process. Consequently, the characteristics and quality of these images, which may vary in configuration and parameters, are crucial in determining the effectiveness and accuracy of the 3D modeling process. According to Hafeez et al. (2018), the comprehensiveness and precision of the resultant 3D models, facilitated by SfM, are intricately tied to both the quantity and quality of discernible points of interest within the images. Therefore, the assurance of high accuracy of the derived products requires the set of images to adequately portray the scene under consideration with an elevated level of detail and quality. This requirement becomes more critical in close-range indoor environments, where scene radiometry can change rapidly due to shadows, specular reflections, and non-uniform artificial lighting.

Image quality is inherently related to the lighting attributes of the captured scene, as argued by Dauvin et al. (2018). The authors highlighted the importance of uniform lighting throughout the region of interest in the scene to facilitate high-quality camera calibration, 3D modeling processes, and more detailed photographic capture of the surface of an object or scene. This factor enables the identification of points of correspondence between images and increases the accuracy and stability of the 3D reconstruction. In practical terms, uniform lighting reduces radiometric inconsistencies across image sets, which helps maintain the repeatability of feature detection and matching between views. Therefore, complementing scene capture with adequate lighting is fundamental to improving the effectiveness of element detection for objects characterized by intricate surface detail with variations in texture and tone. In many cases, solar illumination alone may be insufficient for objects with sparsely detailed surfaces or for laboratory experiments conducted under constrained lighting layouts.

Numerous studies in different domains, including those by Capéran et al. (2012), Kwak et al. (2013), Mishra et al. (2017), Nietiedt et al. (2020), and Nielsen et al. (2023) have addressed the challenge of low object detail using artificial textures projected or applied to object surfaces. This approach has yielded remarkable results, enhanced surface detail, and introduced new patterns, thereby facilitating the detection of a larger number of salient points of interest. In the context of this study, these drawing patterns correspond to manually applied surface markings (i.e., surface painting) intended to increase local contrast and feature detectability under controlled indoor acquisition. For consistency with the terminology commonly used in SfM literature, these applied markings are treated as “artificial texture” in the remainder of the paper.

Besides lighting and surface texture, the choice of storage formats is another factor that influences the representation of the imaged object and JPG or JPEG (Joint Photographic Experts Group) format is preferred in widely used capture systems. However, whereas it offers a wide range of colors with minimal storage requirements, its data compression process can result in loss of information or reduced image quality. Such data loss adversely affects the image correlation processes that are essential to the effectiveness of the SfM technique. Even when lossless options are used, differences in radiometric representation between formats may influence the detection of subtle image features, particularly in precision-oriented close-range workflows.

To address the quality degradation caused by JPG, some professionals have used photographic equipment that produces uncompressed digital images, known as RAW files. Such files preserve the fidelity of the data captured by the camera sensor, enabling a more accurate representation of the scene. However, the superior quality of RAW images, often stored in TIFF (Tagged Image File Format), results in significantly higher storage space requirements compared to JPEG. Morgan et al. (2017) and Verma and Bourke (2019) investigated those file formats. The former study reported consistency in model quality between them; however, the raw format offered advantages in facilitating extensive image processing due to a richer data representation. Analyses conducted in the latter study revealed a slight superiority in positional accuracy of models generated in the raw format attributed to enhanced detection of salient points of interest. Notably, a more detailed scene representation can potentially yield more effective and higher-quality 3D models. Therefore, selecting the storage format involves a practical trade-off between potential gains in reconstruction robustness and the additional computational and storage costs associated with higher-fidelity files.

## Methods

In this study, we utilized Agisoft Metashape Pro ( www.agisoft.com) for 3D processing, a commercial software extensively cited in the literature. Alternatively, open-source software options such as Meshroom ( alicevision.org), OpenMVG (openmvg.readthedocs.io), and COLMAP (colmap.github.io) can also be employed for 3D model generation using SfM techniques. The choice of Metashape Pro does not affect the generality of the conclusions, as the present study focuses on acquisition-stage variables rather than software-specific implementations.

The 3D reconstruction workflow was processed in Agisoft Metashape Pro using a fixed and consistent configuration for all datasets to ensure comparability between experiments. Photo alignment was performed with **High accuracy**, a **key point limit of 60,000**, and **no limit** applied to tie points. Dense point cloud generation was carried out using **High quality** with **Aggressive depth filtering** to reduce noise while preserving geometric detail. Mesh reconstruction was performed with **High quality**, **High face count**, and **Aggressive depth filtering**. Additionally, tiled model generation followed the same configuration, using **High quality**, **High face count**, and **Aggressive depth filtering**. No manual optimization or dataset-specific parameter tuning was applied, ensuring that observed differences in model quality were attributable to acquisition conditions rather than processing adjustments.

The primary aim of the research is to enhance modeling accuracy by optimizing parameters during the photographic acquisition phase. Consequently, the workflow and software configurations are not discussed in detail within this paper. For more information on configurations, of the workflow of the technique, see Leon *et al.* (2015), James *et al.* (2017), and Tinkham and Swayze (2021). This approach allows the analysis to concentrate on capture conditions, which constitute the central scope of this investigation.

### Evaluated test objects

The photographs were taken in the Geomatics Laboratory of the Transport Engineering Department of the São Carlos School of Engineering at the University of São Paulo. The selected test site has both natural and artificial lighting, which, according to the objectives of this study, influenced the photographic capture settings. All experiments were conducted indoors under controlled conditions to reduce external environmental variability.

Samples of three materials, namely concrete, metal, and wood, simulated elements commonly used in laboratory testing of structural beams. The dimensions of the objects were 140 cm × 30 cm × 4 cm for the concrete beam, 140 cm × 40 cm × 1 cm for the metal structure, and 140 cm × 18 cm × 4 cm for the wooden beam.

The regions of interest for each object were defined as the surface planes of the respective objects in the captured photographs. Eight sets of acrylic rulers with checkerboard patterns were randomly placed around these regions to facilitate the automatic detection of reference points by the modeling software.

**Figure 2.**
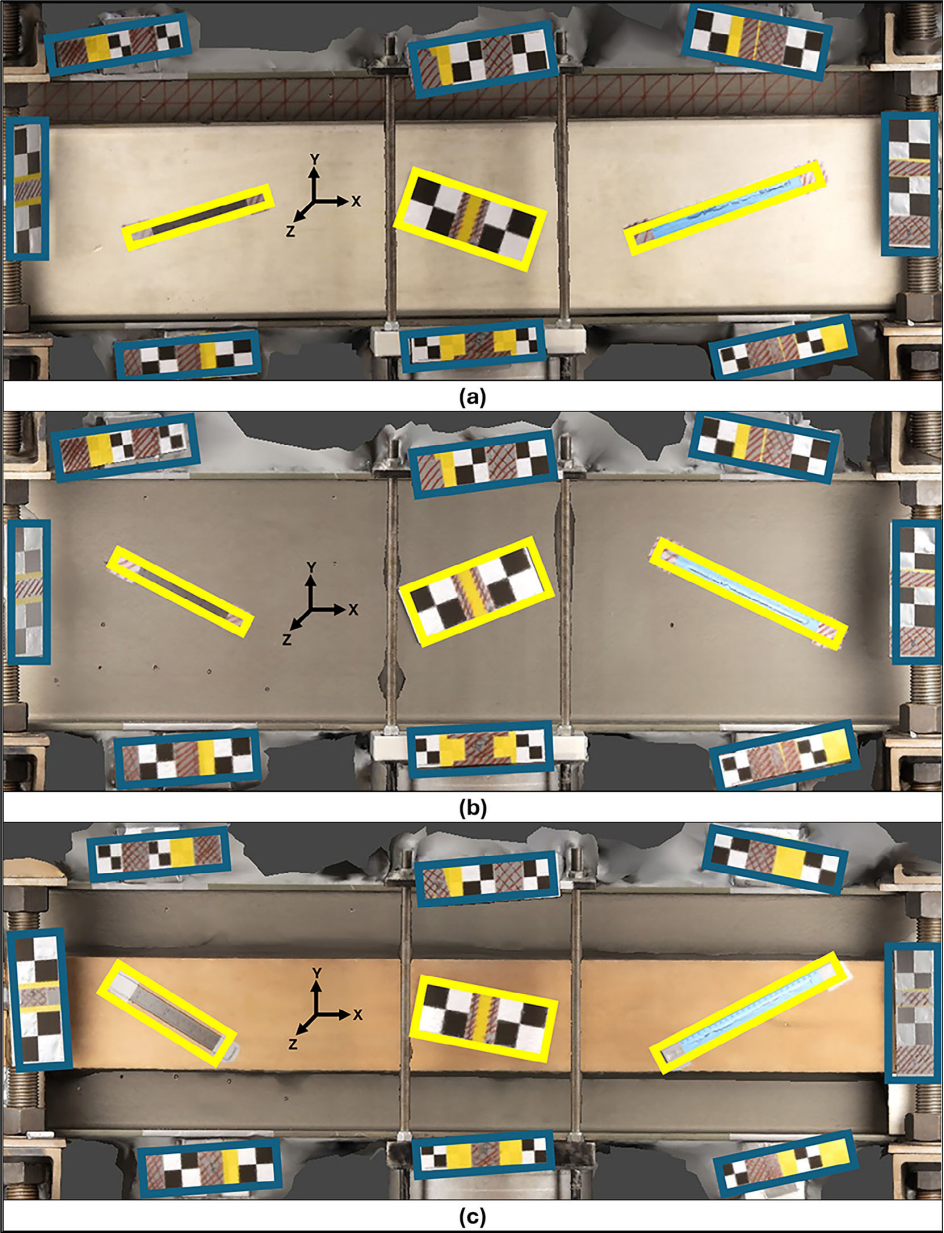
Layout of the arrangement of positional elements, with Scale Bars in blue and Control Bars in yellow for (a) Concrete, (b) Metal, and (c) Wood.

These acrylic plates of known dimensions functioned as scale bars for calibrating and scaling the resulting 3D models. Another set of three acrylic rulers, to be used as control bars (CBs), with checkerboard patterns and known dimensions, were placed within the region of interest of the test specimens to verify the quality of the 3D modeling. The bars were placed at different lengths and positions relative to the axes of the specimens being analyzed. The number, geometry, and placement of scale and control bars were kept constant across all experiments to ensure comparability between configurations. Figure 2 shows the regions of interest and the configuration of SBs and CBs for each material.

Because of its significant dimensions and weight, a robust support structure was necessary to ensure the metal specimen’s stability and safety during imaging. This arrangement resulted in the support structure and the metal specimen being fixed in the center of the room. Consequently, the other specimens were placed next to the metal specimen, creating a consistent environment for analyzing all variables in this research.

### Positioning of auxiliary lighting

In laboratory tests, the standard lighting of the location can cause variations in the color of the same region in different images due to the internal environmental conditions of the facilities. Farhadmanesh *et al.* (2021) addressed how such variations in representation can negatively affect the effectiveness of the interest point detection (SIFT-like algorithms), as discussed in Badano *et al.* (2015) and Lurie *et al.* (2017).

**Figure 3.**
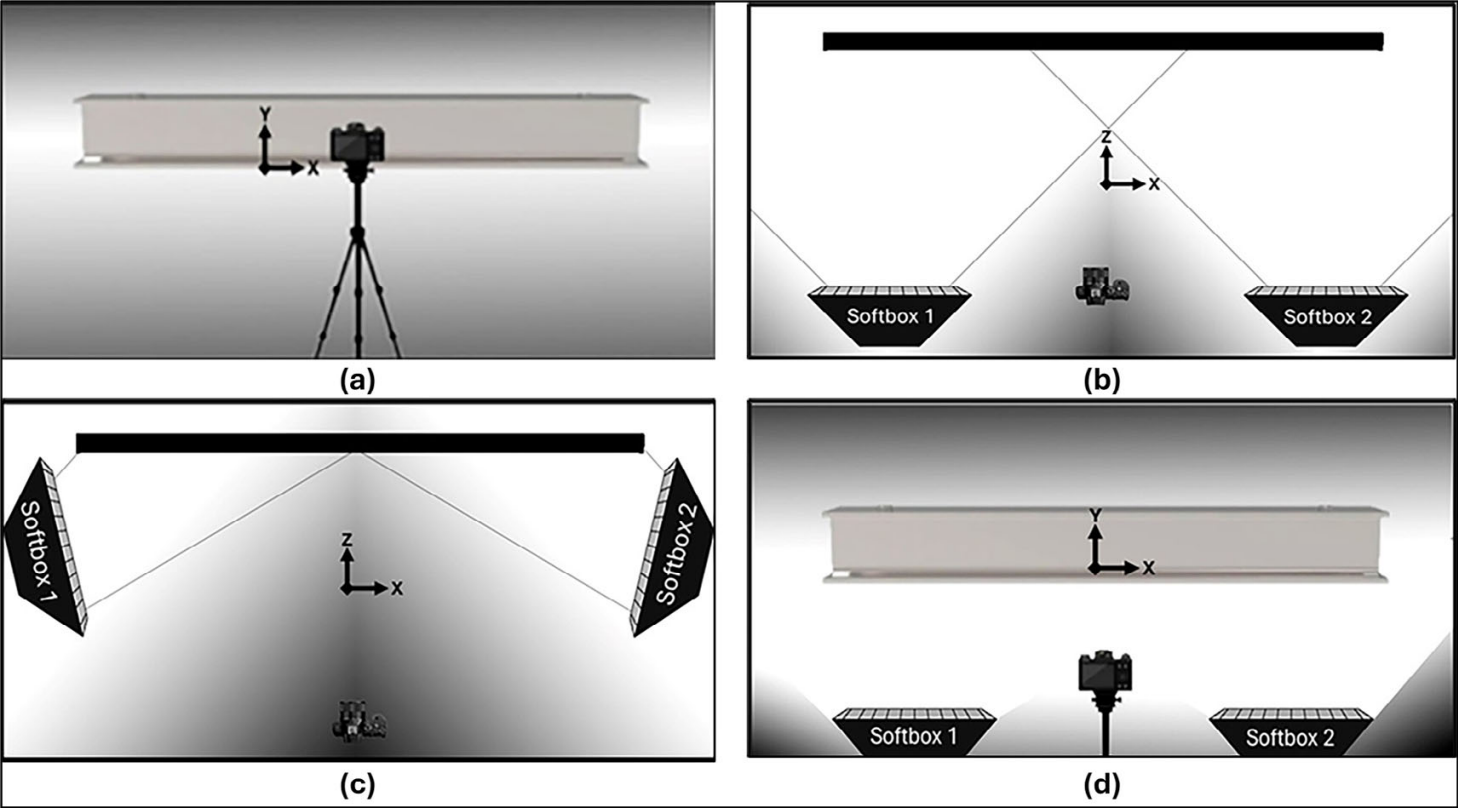
Lighting Equipment Positions: (a) Standard configuration (b) Vertical alignment with the specimen along the camera capture line, (c) Adjacent placement on the sides of the object, and (d) Positioning below the object.

To meet the light quality requirements of this study, two auxiliary lighting units (softboxes) were used, each with a 7,000-lumen LED lamp and a color temperature of 5,000 Kelvin. The softboxes were arranged in three different positioning configurations relative to the specimen to simulate different structural test environments (see Figure 3).

The initial “Standard” configuration ( Figure 3a) depicts the environment in its natural state, utilizing only the room’s ceiling lighting and without any additional light sources. In the “Vertical” model ( Figure 3b), auxiliary lighting was strategically positioned along the camera’s line of sight and directed towards the specimen to illuminate the object vertically and avoid shadowing caused by the acquisition process.

In the “Adjacent” configuration ( Figure 3c), softboxes were positioned in the lateral regions of the test object, simulating tests where other equipment is required between the camera and the object to avoid shadowed areas due to obstruction of lighting.

Finally, the “Beneath” configuration ( Figure 3d) shows an arrangement in which softboxes need to be positioned below the object for replicating experimental environments with space limitations for positioning lighting sources in “Vertical” or “Adjacent” configurations relative to the object. These configurations were selected to reflect realistic laboratory constraints encountered during structural testing.

Reconstruction sets were generated for each lighting scenario on multiple specimens to evaluate the impact of the proposed lighting configurations on the quality of the resulting 3D models. Given that the primary objective of this study is to enhance the image capture process, no post-processing techniques were employed to adjust colors or lighting. This ensured that observed differences in model quality were attributable exclusively to acquisition conditions.

This approach was chosen to ensure proper light exposure of the objects and environment, thereby facilitating the capture process and producing images rich in detectable and correlated features for the SfM and Multi-View Stereo algorithms, as discussed in O’Connor (2018) and Pena-Villasenin *et al.* (2019) without relying on labor-intensive post-processing steps.

The combinations utilized are presented in Table 1, which highlights the adopted lighting configurations, and the number of images generated at each acquisition stage.

**Table 1.** Processing combinations for various materials and lighting configurations, detailing image quantities obtained.

ID	Material	Lighting model	No. of images
CNA	Concrete	Standard (A)	42
CNB		Vertical (B)	
CNC		Adjacent (D)	
CND		Beneath (E)	
MNA	Metal	Standard (A)	42
MNB		Vertical (B)	
MNC		Adjacent (D)	
MND		Beneath (E)	
WNA	Wood	Standard (A)	42
WNB		Vertical (B)	
WNC		Adjacent (D)	
WND		Beneath (E)	

### Artificial texture patterns

Artificial texture patterns were applied to the surface of the specimens to enhance the detection of points of interest in the materials used, highlighting details of natural texture and color contrast on the object, thus facilitating the detection and correlation of these points between photographic sets. In this study, the term “artificial texture” refers exclusively to manually applied surface drawing or painting patterns used to increase local visual contrast. No projected patterns, structured light systems, or active texture generation techniques were employed.

**Figure 4.**
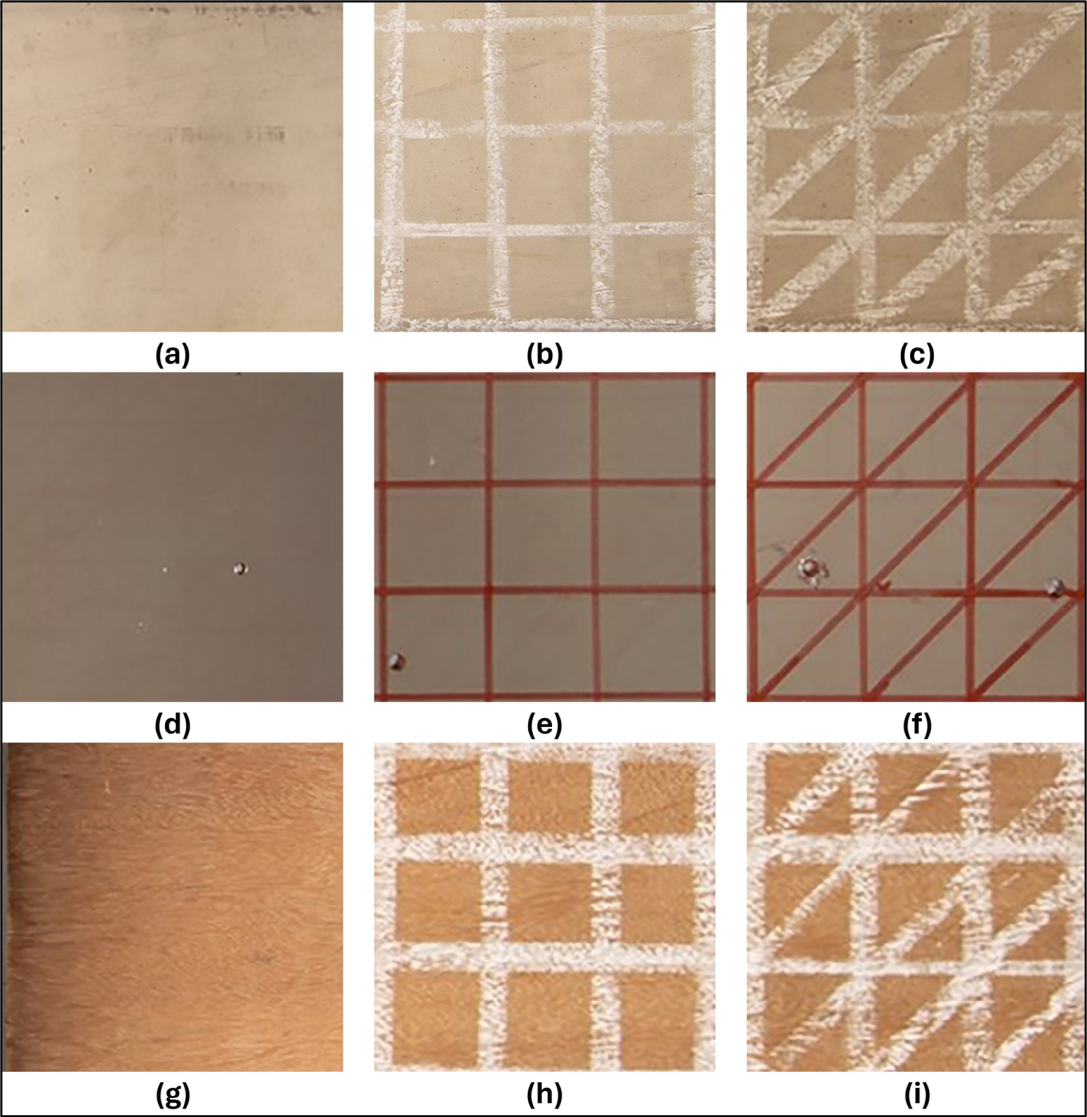
Natural and artificial textures associated with each specimen analyzed. (a) Natural texture of the concrete; (b) Artificial texture T1 in concrete; (c) T2 artificial texture on concrete; (d) Natural texture of the metal; (e) T1 artificial texture on metal; (f ) T2 artificial texture on metal; (g) Natural texture of the wood; (h) T1 artificial wood texture; (i) T2 artificial wood texture.

In Reiss and Tommaselli (2011) and Detchev *et al.* (2014) image projectors were used to create random patterns on the surfaces of the analyzed objects. However, in the present study, due to the use of reference bars with checkerboard patterns and the analysis of the use of auxiliary lighting, image projection would introduce challenges in the automatic detection of reference bars and would impact the lighting configurations of the environment. Therefore, two random patterns, a checkerboard pattern (T1) and a more complex pattern (T2), were drawn on the surface of the objects. Figure 4 shows the sets of natural textures of the samples and the artificial patterns used.

Due to the rich surface texture of the concrete and wood samples, a 4 cm x 4 cm checkerboard pattern (T1) drawn in white chalk and a second pattern (T2) of diagonally cut squares were used. The metal object analyzed, on the other hand, had a more uniform surface due to the texture of the material and the primer applied to protect it from corrosion. A red permanent marker was used to accentuate the color of the metal sample using the above drawing patterns.

The use of white chalk for the concrete and wood specimens, along with a red permanent marker for the metal specimen, was strategically chosen to create a high contrast between the colors of the test specimens and the artificial texture patterns. This deliberate contrast, coupled with the varied pattern representations on the object surfaces and precise exposure to auxiliary lighting, is designed to optimize the detection of a substantial number of elements by SIFT-type algorithms as discussed in Kanan and Cottrell (2012) and Wang *et al.* (2023).

### Data acquisition and storage formats

In the SfM, the photo acquisition stage is key in producing high-quality 3D models ( Caldera-Cordero and Polo 2018). Accurate image capture is essential for ensuring precise alignment, detailed reconstruction, and reliable measurement outcomes, particularly in structural analysis and laboratory testing scenarios.

The photographic capture process for this study incorporated scale bar (SB) configurations, image overlap percentages, and camera calibration techniques to facilitate the analysis of the relevant variables. These configurations and processes align with the methodologies outlined in de Moraes and da Silva (2024).

**Figure 5.**
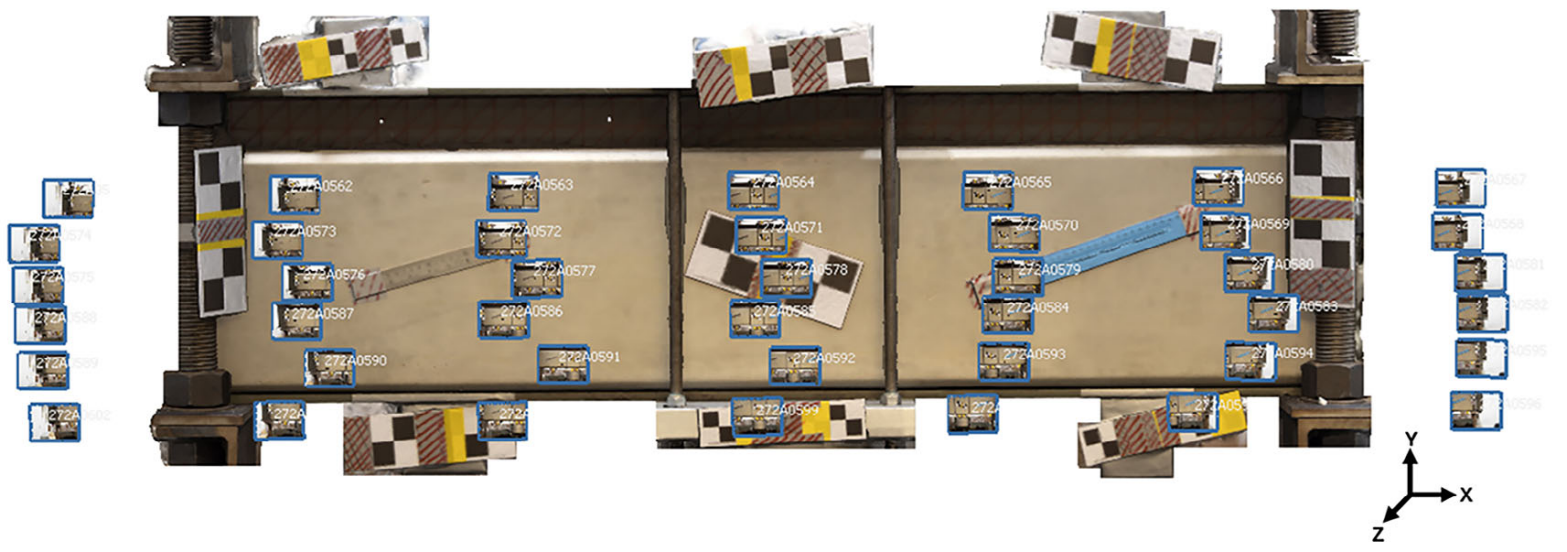
Photographic capture process of the concrete specimen highlighting the blue squares that symbolize each image acquired during the procedure.

For each analysis, images were acquired following an ordered variation in camera positioning to establish a capture method like a regular vertical grid model. Figure 5 shows the photographic capture process for the region of interest on the concrete object using only vertical captures.

A full-frame Canon EOS R camera, paired with a Canon RF 24-105 mm f/4L zoom lens, was employed for the photographic process. The camera was configured to manual mode, with a fixed focal length of 35mm to ensure consistent settings across all image captures. Exposure compensation was adjusted to +1 EV to enhance the brightness of all captures. This setup provided precise control over exposure parameters and captured regions with sufficient detail to meet the submillimeter precision requirements of the study.

To minimize image noise and ensure a greater depth of field, each shot was taken at ISO 100 and an aperture of f/11 was selected to improve focus across the scene, ensuring sharpness across the entire field of view. To further ensure the accuracy and stability of the images, a tripod, and a 5-second timer shutter were used.

A 1-meter capture distance was adopted in the experiments to evaluate the SfM technique used in structural testing. This distance maintains Ground Sample Distance (GSD) values and provides a more efficient capture process. The GSD values obtained for the experiments were approximately 0.15 mm. The image settings were configured to 6,720 x 4,480 pixels, with a 35 mm focal length on a camera equipped with a 35 mm × 24 mm full-frame sensor. This distance was selected to balance safety constraints and the need for submillimeter modeling accuracy.

The photographic images were in RAW format. However, the image sets were converted to JPG and TIFF formats to evaluate the impact of different storage formats on the quality of 3D modeling. According to Detchev *et al.* (2014), Morgan *et al.* (2017) and Verma and Bourke (2019), these two formats are widely used in projects employing SfM. We chose the commercial software Adobe Photoshop ( www.adobe.com/br/products/photoshop.html) to convert the raw files captured by the camera to TIFF and JPG formats, because it is easy to use. However, open-source computer applications such as RawTherapee ( www.rawtherapee.com), darktable ( www.darktable.org), and GIMP ( www.gimp.org) also successfully meet our needs.

A Starrett EC799A-8 digital caliper was used to measure the lengths of the sets of SBs and CBs to size and check the 3D models developed. The instrument accurately checked the distances between the markings on the rulers and the targets of the reference elements to sub-millimeter accuracy. It offers a prominent level of precision, with error margins of ± 0.02 mm for measurements up to 10 cm and ± 0.03 mm for measurements above 10 cm Company (2007).

**Figure 6.**
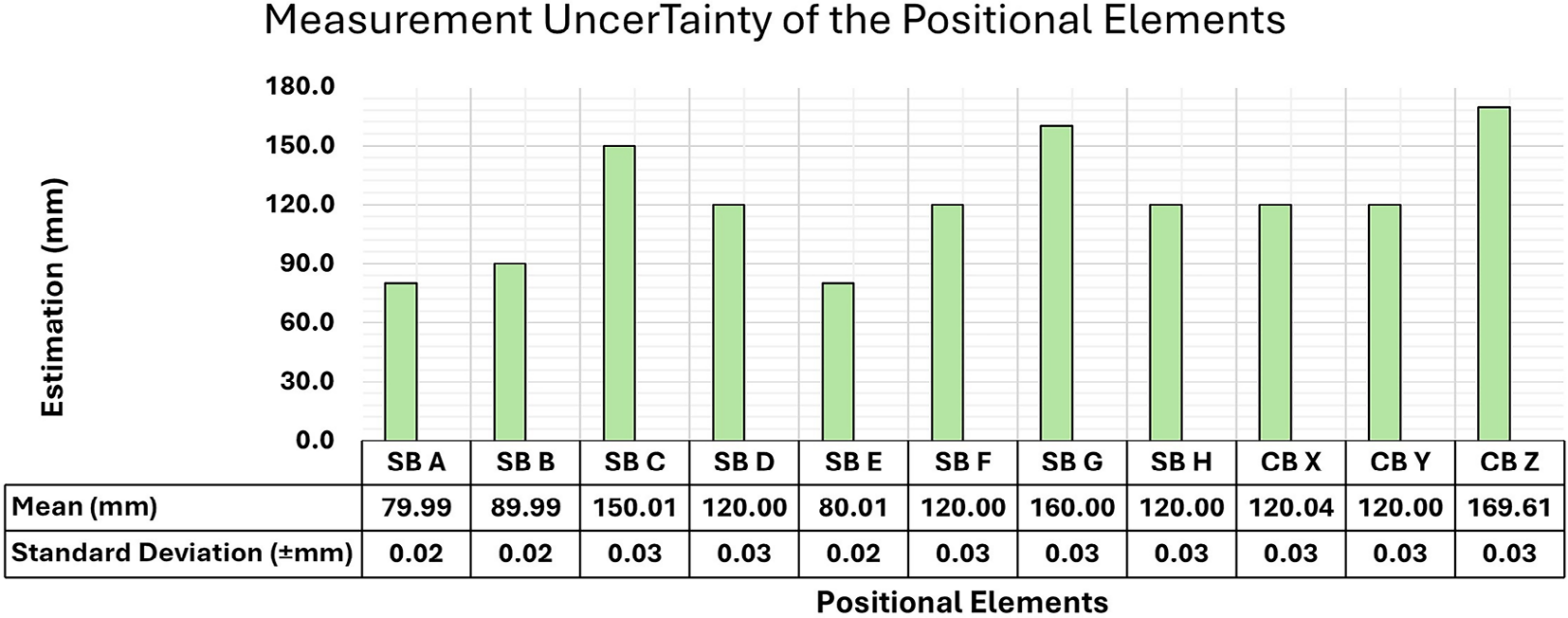
Representation of the average lengths and Standard Deviation of the measurements of each positional element obtained from five measurements.

Five measurements were taken for each positional element to determine the length of the SBs and CBs accurately. The accuracy of the reference elements, combined with the positional accuracy of the SfM modeling, played a crucial role in determining the accuracy values used in the evaluation of the developed models. Figure 6 shows the mean values of the length of each bar obtained from the set of measurements, together with the standard deviation for each positional element. All measurements of the bars and images utilized in this study are freely accessible in the OSFHome repository at the following link: https://osf.io/k82ar/.

### Quality assessment

Initially, from a set of input images, the SfM process generates a point cloud referenced to the camera coordinate system, resulting in an inaccurate representation of real-world objects. Therefore, a scaling procedure is essential to scale the point cloud relative to a specific reference unit and ensure the positional accuracy of the three-dimensional product ( Luhmann et al. 2020). Different formats and positioning configurations of SBs with known lengths were used to scale and refine the generated products.

A series of procedures were applied to evaluate the length of the positional elements used to estimate the positional accuracy of the models. First, the lengths were obtained by measuring the generated virtual models and compared with the values obtained from the digital caliper measurements. As discussed by Garcia and Oliveira (2021), the RMSE value ( equation 1) was used to analyze the error and evaluate the accuracy of the distance prediction.

$$\text{RMSE}=\sqrt{\frac{\sum_{i=1}^{n}{\left(E_{i}-R_{i}\right)}^{2}}{n}}$$ (1)

where *n* is the number of samples, *E*_*i*_ is the estimated value at position *i*, and *R*_*i*_ is the value measured at position I.

Together, these metrics enabled a comprehensive assessment of reconstruction accuracy and robustness across different acquisition configurations.

## Results and discussions

This section provides the results and discusses the experiments conducted.

### Assessment between different lighting configurations

**Figure 7.**
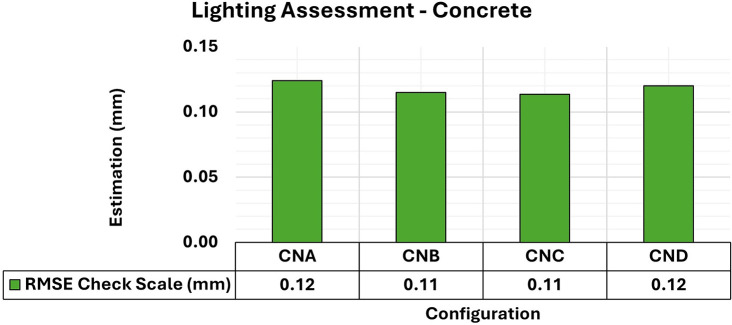
RMSE values of CBs for different lighting configurations in a concrete specimen.

**Figure 8.**
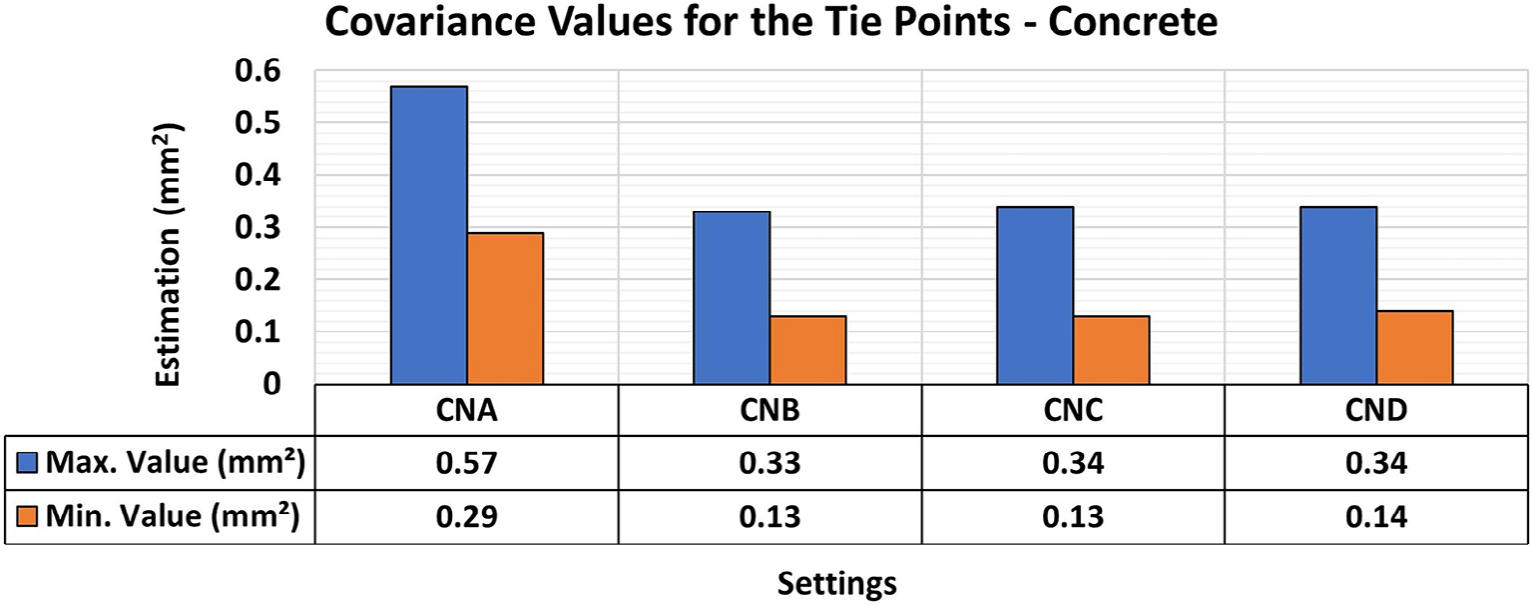
Maximum and minimum values from the Variance and Covariance Matrix indicate the quality of adjustment for each set of 3D modeling of the concrete specimens, with best results in the CNB configuration - maximum value of 0.33 mm ^2^ and minimum one of 0.13 mm ^2^.

Image sets with an overlap of approximately 80% were used to generate different 3D models. Camera calibration parameters were obtained using the pre-self-calibration method, using the maximum number of SBs (8) in environments like the region of interest, with consistent lighting configurations for each analysis. Figures 7 and 8 show the results of the RMSE values for these configurations in the concrete samples and the maximum and minimum diagonal values of the variance and covariance matrix. In this study, RMSE values computed from control bars (CBs) represent positional accuracy, while the maximum and minimum diagonal values of the variance–covariance matrix are used as indicators of adjustment stability and internal precision of the reconstruction.

Consistent RMSE values ( Figure 7) between 0.12 mm and 0.11 mm were observed for the positional quality of 3D modeling for the different lighting setups in the concrete specimens. However, in terms of the quality of the modeling adjustment ( Figure 8), a significant improvement was achieved when auxiliary lighting was used in the photographic capture process compared to the natural lighting configuration (CNA). The values averaged 0.34 mm ^2^ and 0.13 mm ^2^ for the maximum and minimum values of the variance and covariance matrix, respectively when additional lighting was used, whereas the natural configuration gave significantly higher results.

These patterns, which indicate an improvement in RMSE values and the quality of 3D model adjustment when additional lighting was used, are attributed to the spectral characteristics of the material used as the test specimen. According to Senevirathne et al. (2021), concrete materials are influenced by factors such as mixture composition, texture, and surface color of the object, leading to a higher reflectance rate and hence more accurate 3D modeling of objects even under limited lighting conditions, as observed in the experiments.

As photographic capture relies on the amount of light reflected from objects to ensure a clearer process, the generated models showed a higher quality of adjustment under higher lighting intensities (e.g. in CNB, CNC, and CND configurations), due to the greater detail of the analyzed objects and areas of interest displayed in the image sets from these configurations, resulting in more accurate modeling.

**Figure 9.**
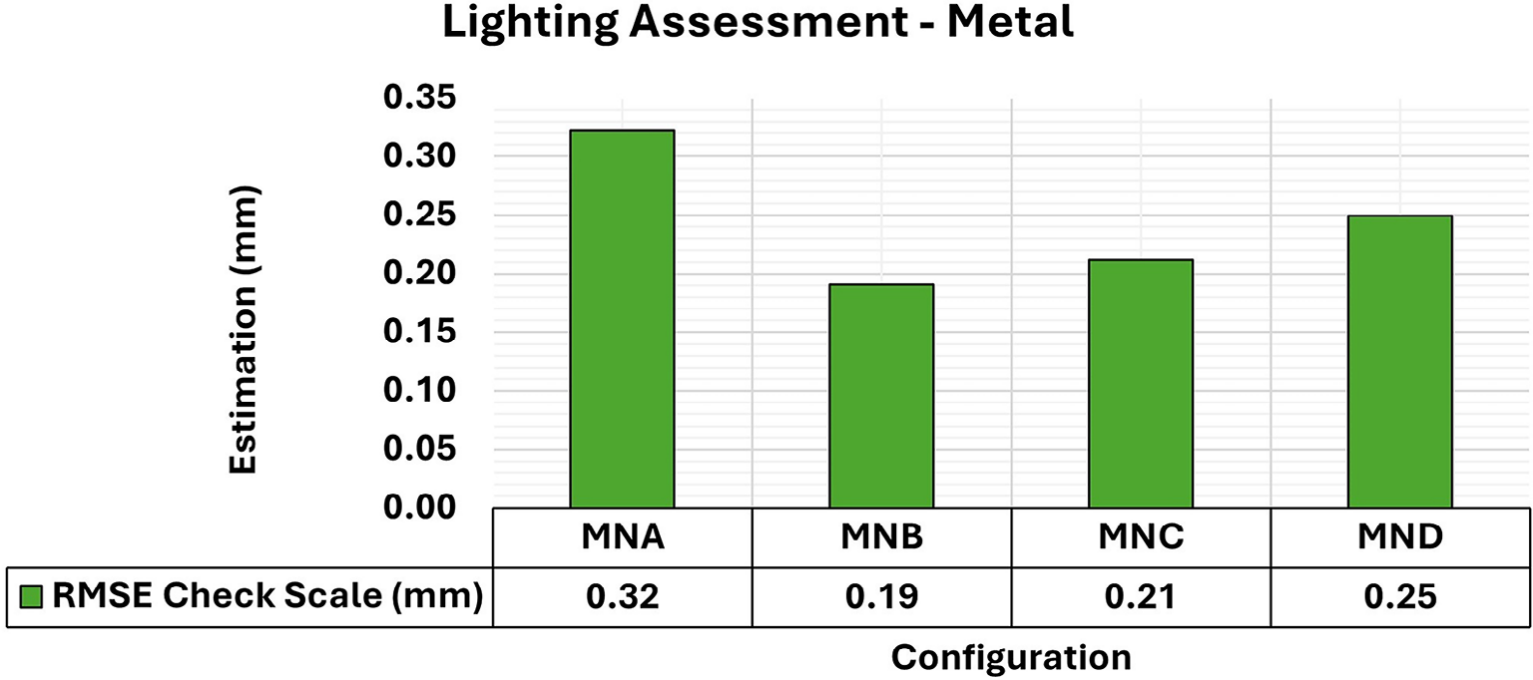
RMSE values of CBs under various lighting conditions in a concrete sample. The assessment yielded values ranging from the least favorable (0.32 mm) in MNA to the most favorable (0.19 mm) in MNB.

**Figure 10.**
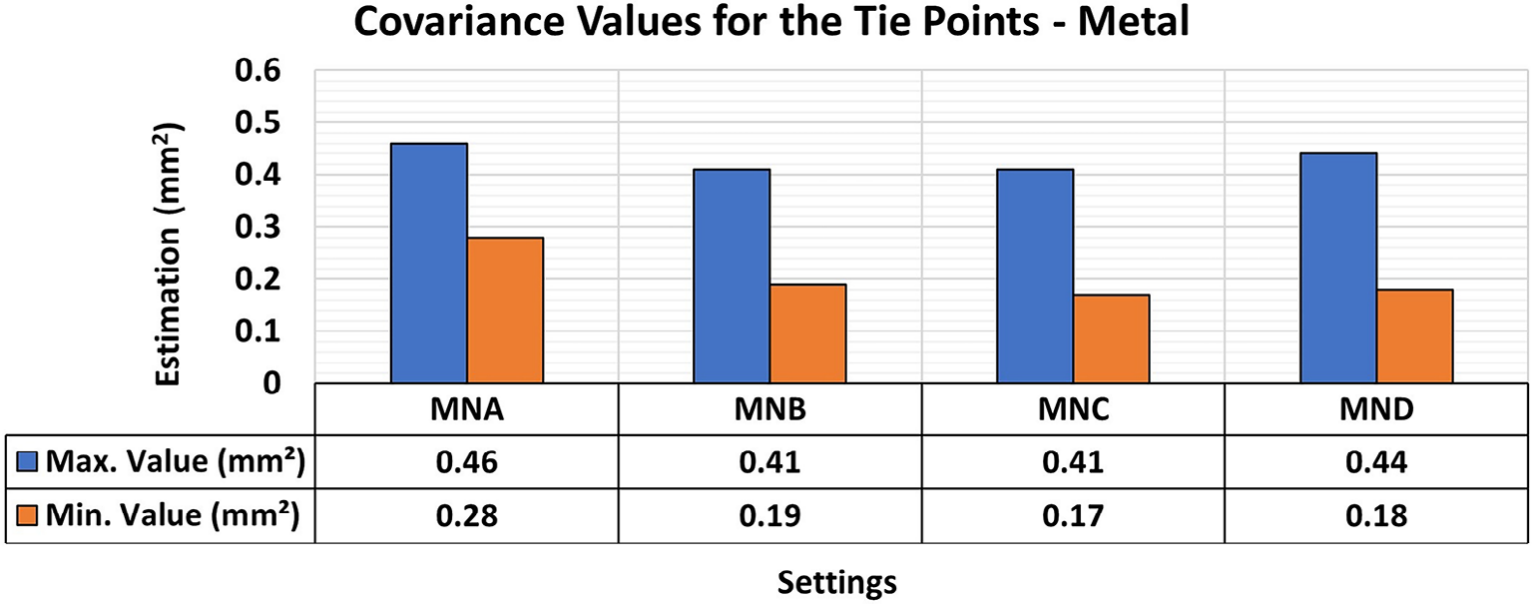
Maximum and minimum values from the Variance and Covariance Matrix indicate the quality of adjustment for each 3D modeling set. Superior results were achieved for the concrete object, with 0.41 mm ^2^ maximum value and 0.19 mm ^2^ minimum one.

Figures 9 and 10 show the results for RMSE and maximum and minimum values of the variance and covariance matrix, respectively, for the different lighting configurations on metallic samples.

The application of a layer of protective paint to the surface of the object is common due to the characteristics of metallic specimens concerning the oxidation process and rust formation. Although the paint protects the metal from corrosion effects, as a side effect it also smoothens the surface of the analyzed object, further reducing the textural properties of the specimens, as discussed by Sudarsanan *et al.* (2019). This smoother surface reduces natural visual texture, which makes feature detection and matching more sensitive to illumination conditions.

The experiments showed a slight improvement in positional quality when any form of additional lighting (MNB, MNC, and MND) was used compared to its non-use (MNA). The RMSE values for the use of additional lighting ranged from 0.19 mm to 0.25 mm and were 0.32 mm when the MNA setup was used.

Regarding the maximum and minimum values of the variance and covariance matrix for the different lighting configurations of metallic objects, a significant improvement was obtained when any form of lighting assistance (MNB, MNC, and MND) was used. The elements of the variance and covariance matrix showed maximum values of the order of 0.4 mm ^2^, which is like that obtained when no additional lighting (MNA) was used. However, compared to the standard model of the test specimen, the minimum values improved by about 0.1 mm ^2^ when any lighting aid was used. This result suggests that auxiliary lighting primarily improves the stability of the adjustment (internal precision), even when the RMSE gains are moderate.

**Figure 11.**
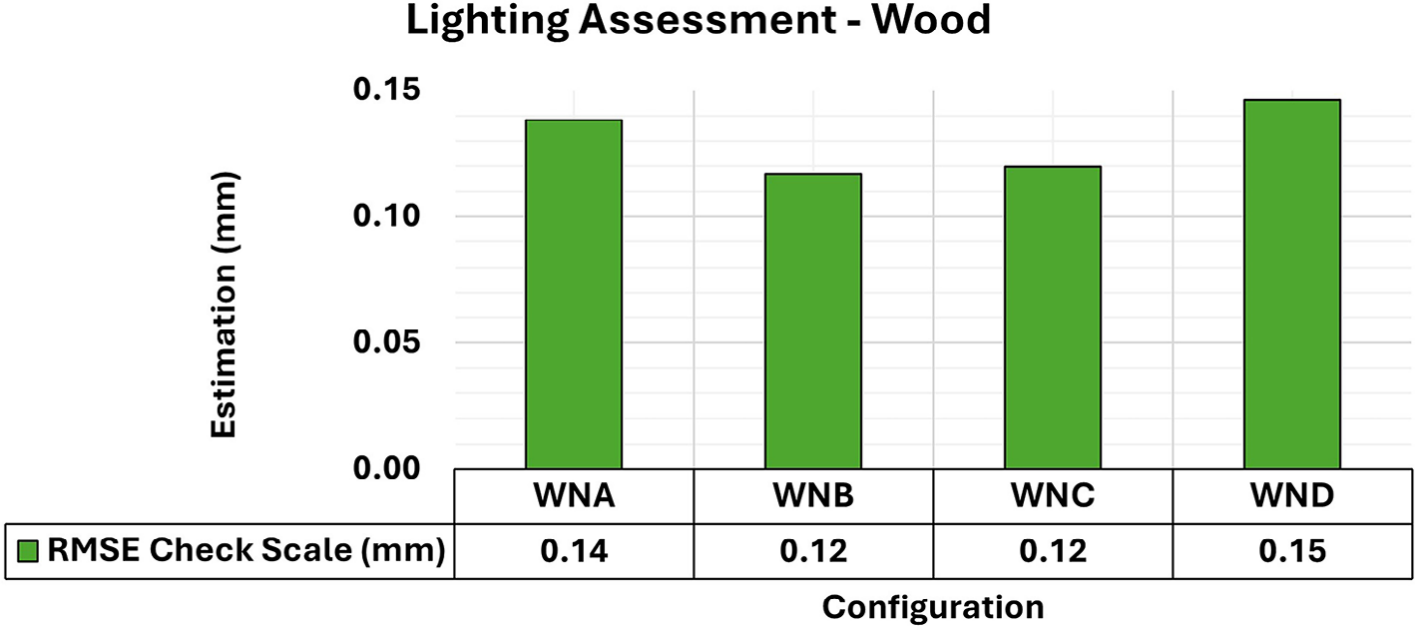
RMSE values of CBs under various lighting conditions in a concrete sample. The assessment yielded values ranging from the least favorable (0.32 mm) in MNA to the most favorable (0.19 mm) in MNB.

**Figure 12.**
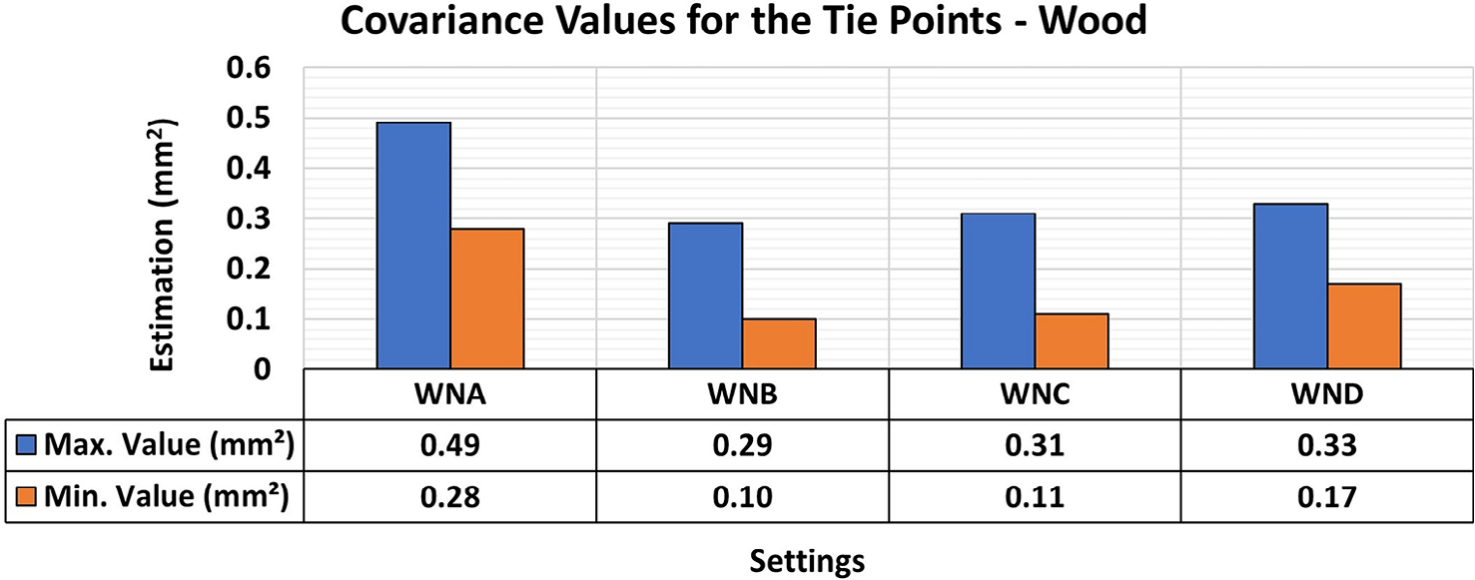
Maximum and minimum values from the Variance and Covariance Matrix indicate the quality of adjustment for each 3D modeling set. Superior results were achieved for the concrete object, with 0.41 mm ^2^ maximum value and 0.19 mm ^2^ minimum one.

Finally, the results of the 3D modeling of the wooden specimens provided RMSE values and maximum and minimum values of the variance and covariance matrix as shown in Figures 11 and 12, respectively.

The RMSE values of the positional quality of the modeling across the different lighting arrangements in the wooden test bodies showed uniformity, ranging between 0.12 mm and 0.15 mm. Additionally, the quality of adjustments improved when any form of auxiliary lighting (WNB, WNC, and WND) was used in the photographic capture process, compared to the natural configuration (WNA).

The results of the quality analysis of the experiments with the wooden specimen can be attributed to the different structural characteristics of the materials. As discussed by Feng *et al.* (2019), the texture of wood shows a wide range of color variations and patterns that can coexist in a single artifact, allowing for detailed photographic capture comparable to that observed in concrete objects. The variety of texture and contrast of the objects’ surfaces facilitated a more detailed capture process, particularly when appropriate lighting was used, improving the recognition of elements in the image sets used and resulting in highly accurate modeling.

**Figure 13.**
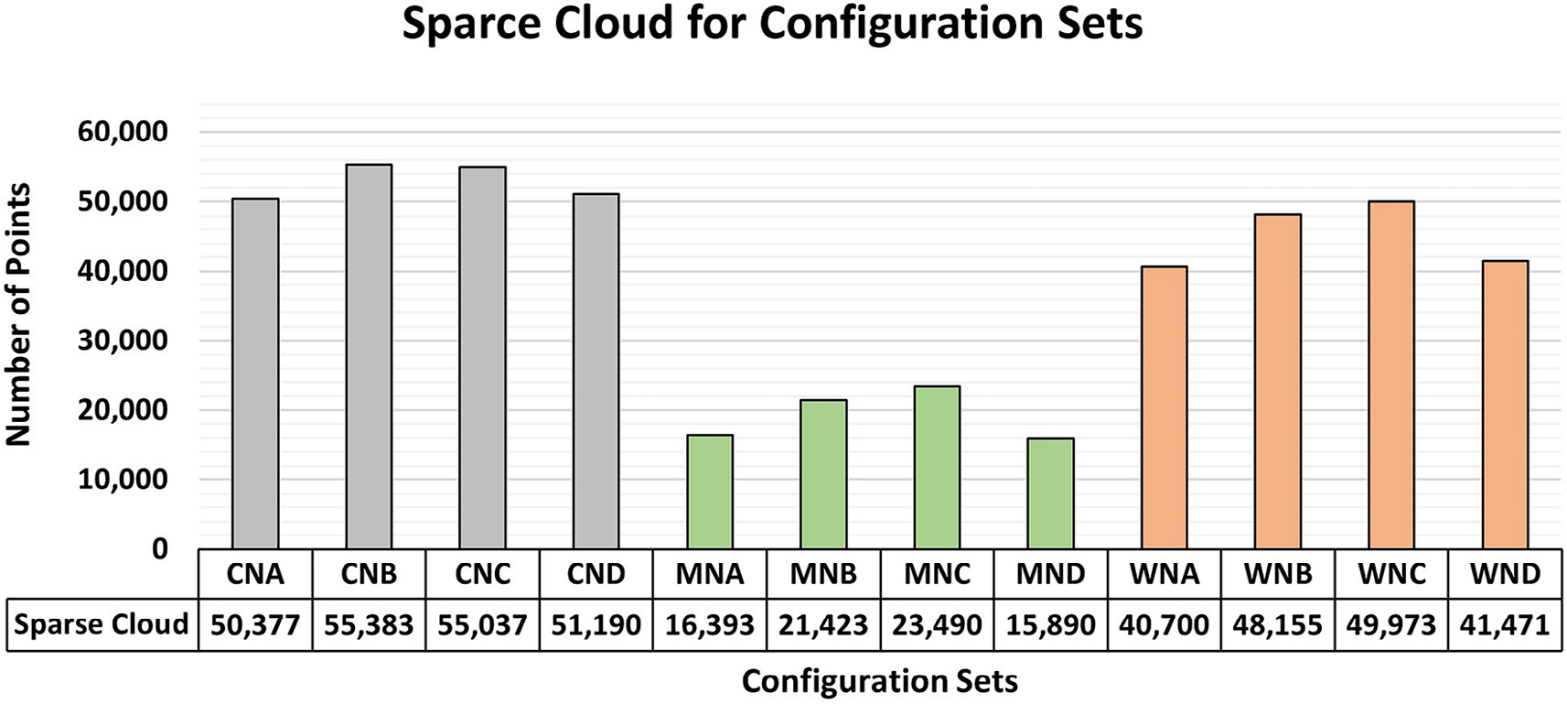
Values related to the sparse point cloud for each material under different lighting configurations employed in photographic capture. Data suggest a slight superiority of Vertical and Adjacent lighting configurations across the three materials studied.

The analyses revealed the integration of lighting assistance substantially improved the quality of the 3D modeling for the three materials examined. However, the number of elements detected in each set of images, considering the various configurations and materials used, was evaluated towards a more comprehensive analysis for the selection of the most suitable configuration and, hence, optimal results. Figure 13 displays the number of sparse cloud points obtained after the detected elements had been filtered.

The number of points obtained for each lighting configuration shows a subtle consistency among the values for the same material analyzed. However, the most favorable results were obtained using the “Vertical (B)” and “Adjacent (C)” lighting configurations. A comparison of such information with previous analyses showed a slight advantage for these lighting configurations in terms of quality parameters. (Note: the “Adjacent” configuration corresponds to the lateral placement described in the Methods and illustrated in Figure 3c.)

Despite its results with minimal variation, the “Beneath (D)” configuration posed significant challenges to equipment installation and usage within a laboratory setting. Space constraints and safety considerations in structural testing, particularly beneath the test specimens, compromise the practicality of implementing the Beneath (D) lighting configuration. Although the configuration offers advantages, organizations must carefully assess its adoption in terms of safety and test feasibility.

Due to the proximity of the values, auxiliary lighting should be positioned directly in front of the object or adjacent to the region of interest for photographic captures aimed at modeling objects with submillimeter precision.

### Analysis of the use of different artificial textures

New sets of capture and processing involving the application of artificial textures to the specimens were initiated as a function of previous findings that underscored the advantages of “Vertical (B)” and “Adjacent (C)” lighting aids during the photographic capture stages. Each material analyzed in the experiment, namely, concrete, metal, and wood, was examined with two distinct texture patterns (T1 and T2) and the natural pattern inherent to each specimen. In this study, “artificial textures” refer to manually applied drawing/painting patterns introduced to increase local contrast and feature detectability; no projected patterns or structured light systems were used.

**Figure 14.**
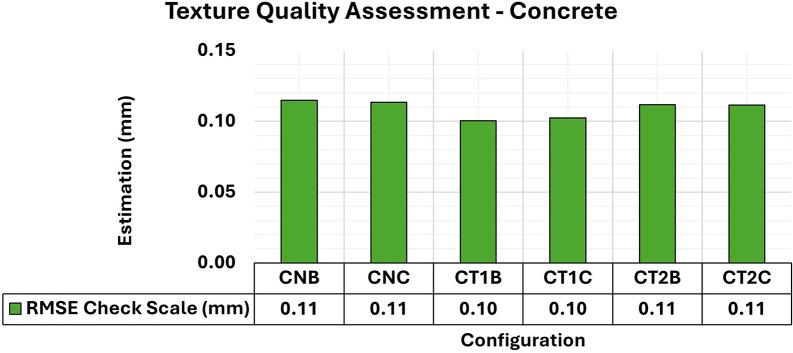
RMSE values of CBs for different texture configurations in a concrete specimen. An approximately 0.11 mm consistency is evident for the assessment.

Figure 14 shows the modeling results of the concrete specimens, in which two different lighting configurations and three texture models were analyzed. The artificial texture models were crafted, in a checkered pattern using white chalk to accentuate details of the surface of the object in the first model and the pattern used in the second.

**Figure 15.**
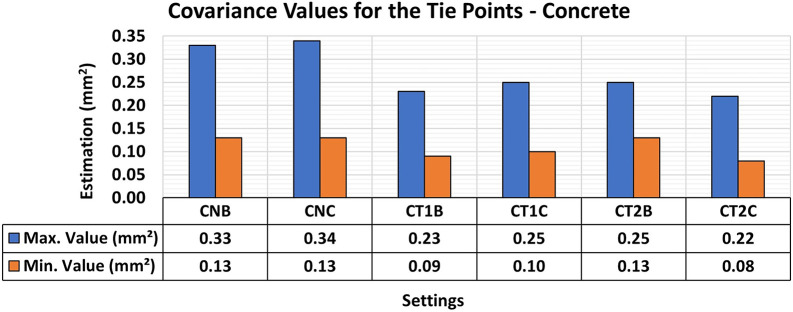
Maximum and minimum values from the Variance and Covariance Matrix indicate the quality of adjustment for each 3D modeling from the concrete set. Superior results were achieved, with 0.22 mm ^2^ maximum value and 0.08 mm ^2^ minimum one.

The results showed a notable equilibrium in the total RMSE values for all configurations, averaging around 0.11 mm. However, the variance and covariance matrix values ( Figure 15) showed differences between the artificial and natural texture models, especially in the maximum values. There was an improvement of approximately 0.1mm ^2^ when artificial texture models were used. This indicates that the applied patterns primarily improved the stability of the bundle adjustment (internal precision), even when positional accuracy (RMSE) remained nearly unchanged.

**Figure 16.**
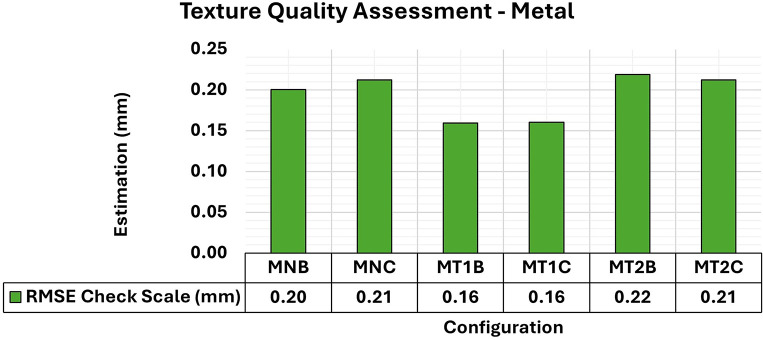
RMSE values of CBs for different texture configurations in a metal specimen. The assessment yielded values ranging from the least favorable (around 0.21 mm) in MT2B and MT2C to the most favorable (0.16 mm) in MT1B and MT1C.

Figure 16 shows the results of the RMSE quality metrics for modeling metallic samples. Texture patterns were created in the analysis using red permanent markers to enhance the contrast with the color of the protective paint applied to the samples. The first artificial texture (T1) aimed to highlight detail by incorporating a checkerboard pattern on the surface. Conversely, the second model (T2) intensified the pattern introduced by T1.

**Figure 17.**
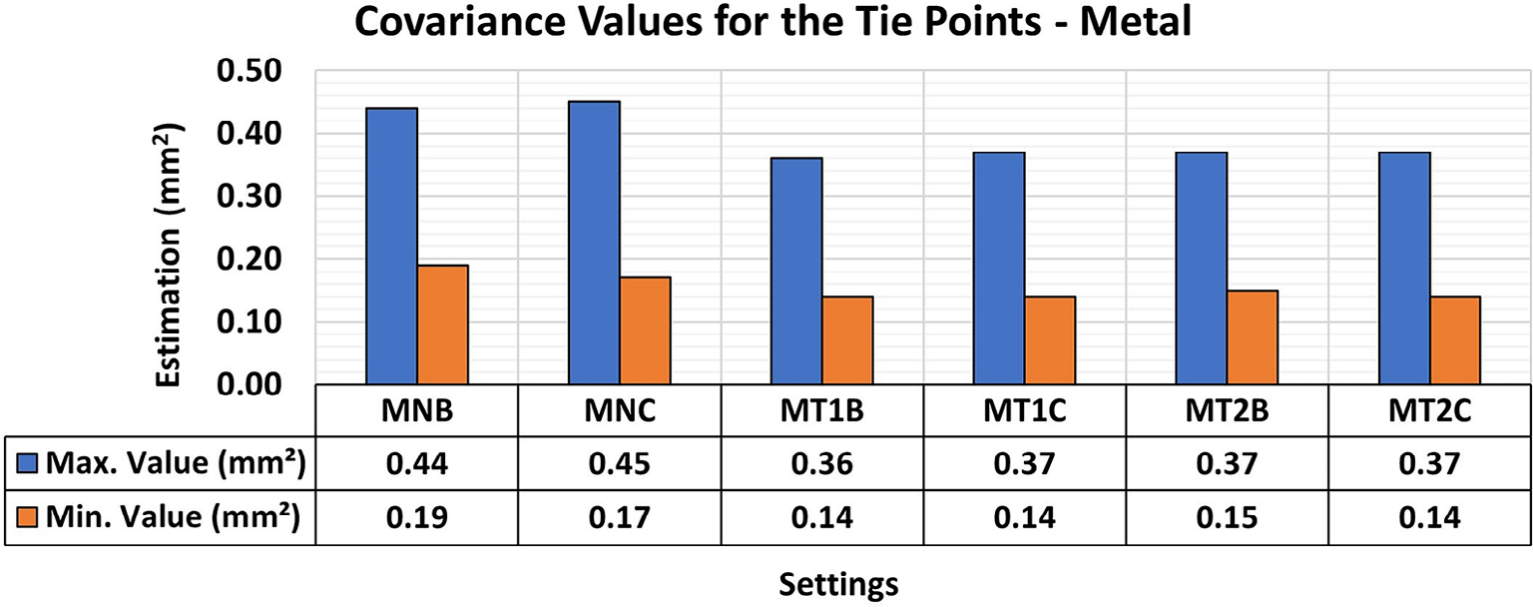
Maximum and minimum values from the Variance and Covariance Matrix indicate the quality of adjustment for each 3D modeling from the metallic set. Superior results were achieved, with 0.36 mm ^2^ maximum value and 0.14 mm ^2^ minimum one.

The T1 texture configuration showed a slight improvement in the RMSE results, with average values of 0.16 mm compared to the other texture configurations, which achieved average values of 0.21 mm. As shown in Figure 17, there was a significant improvement in both the maximum and minimum values of the variance and covariance matrix when each artificial texture model was used. This behavior is consistent with the reduced natural texture of the painted metal surface, for which added high-contrast markings can substantially increase the number and spatial distribution of reliable matched features.

**Figure 18.**
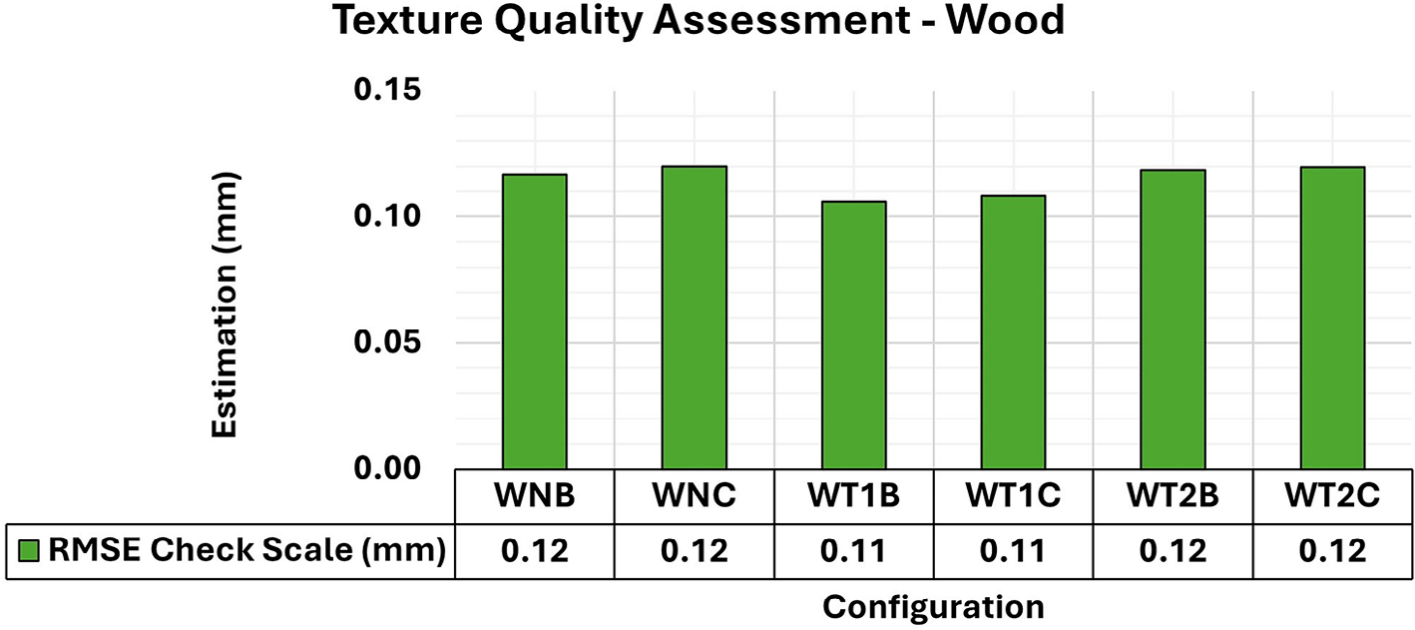
RMSE values of CBs for different texture configurations in a wood specimen. An approximately 0.12 mm consistency is evident in the assessment.

Figure 18 shows the RMSE results of wooden object modeling with configurations of artificial textures like those used in concrete specimen experiments. The artificial texture models on the wooden specimen were created using white chalk in two different patterns to accentuate the surface details of the object. The first pattern (T1) follows a checkered pattern while the second (T2) has a denser pattern.

**Figure 19.**
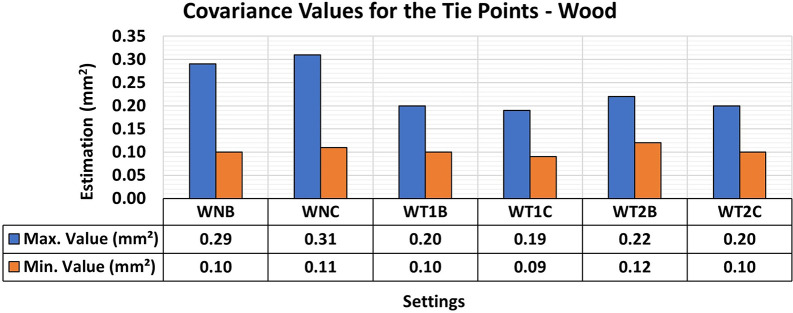
Maximum and minimum values from the Variance and Covariance Matrix indicate the quality of adjustment for each 3D modeling from a wood set. Superior results were achieved, with 0.19 mm ^2^ maximum value and 0.09 mm ^2^ minimum one.

The behavior of the wooden specimens was remarkably like that of concrete objects. Although the RMSE values did not show significant variations with the application of different texture settings, the adjustment accuracy of the 3D models improved. Figure 19 shows the maximum and minimum values of the diagonal of the variance and covariance matrix for modeling the wooden specimen, emphasizing a notable improvement in quality in terms of maximum values when an artificial texture model was applied.

Analysis of the results for the three materials showed similar behavior, despite the distinctive characteristics associated with the natural texture patterns and color of each sample. The use of artificial standards resulted in more accurate 3D modeling with less variation in the maximum and minimum values, as shown by the variance and covariance matrix values. However, no significant gains in modeling were observed when positional quality was analyzed using RMSE values.

The consistency of the positional quality values is due to the image sets used in the analysis. The use of artificial lighting in all analyzed sets resulted in highly accurate modeling, as previously investigated, and combined with artificial textures, led to the acquisition of point clouds with low representation errors, as discussed by Hafeez *et al.* (2018).

The analysis suggests that artificial textures tend to improve the accuracy of 3D object modeling and facilitate the detection of elements between images, resulting in a more detailed representation. The experiments involved the use of light tools and artificial textures with significant contrast to the materials analyzed. It is therefore the responsibility of the user to determine the most appropriate pattern and methods for representing the texture of an object, considering the specific requirements of their experiments in terms of feasibility and potential benefits. In engineering laboratories, particularly for structural testing requiring sub-millimeter precision, the use of artificial textures with checkerboard patterns and a high degree of repetition is recommended to improve the quality of 3D modeling. However, overly dense or highly repetitive patterns may obscure fine surface details of interest (e.g., cracks), and should be selected according to the inspection objective.

### Analysis of different storage formats

Additional 3D modeling processes explored the effects of using different storage formats (TIFF and JPG) in sets of images captured in indoor environments at close range. Artificial textures (T1) and lighting support (B – Vertical) were adopted for the three test specimen materials.

The combinations made, including formats and various configurations, are presented in Table 2, along with the corresponding amounts of storage used by each format. The image sets indicate that the average size of files in TIFF format was approximately fifteen times larger than those in JPG format. This substantial difference highlights the practical implications of storage format selection in large-scale or repeated experimental campaigns.

**Table 2.** Processing combinations for specimen materials, storage formats, and average image size for 3D modeling.

ID	Material	Texture	Lighting model	Format	Size per image (MB)
CT1B – TIFF	Concrete	T1	B	TIFF	84.1
CT1B – JPG				JPG	5.78
MT1B – TIFF	Metal	T1	B	TIFF	84.1
MT1B - JPG				JPG	5.78
WT1B – TIFF	Wood	T1	B	TIFF	84.1
WT1B - JPG				JPG	5.78

**Figure 20.**
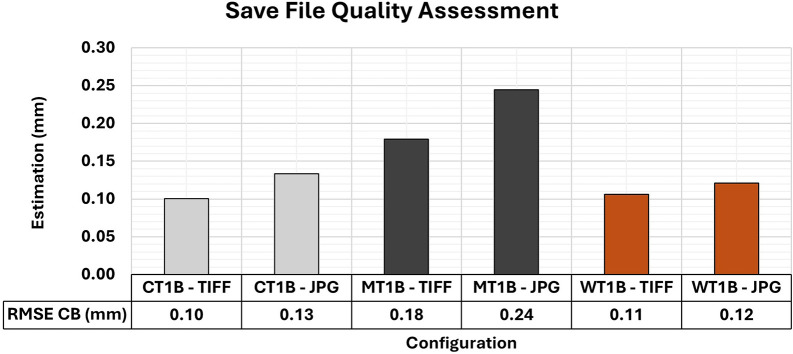
The RMSE values of CBs ranged for different save file configurations (TIFF and JPG) across all materials when artificial texture T1 and lighting condition B (Vertical) were used. The TIFF configuration yielded better RMSE values in the assessment compared to the 3D models with the use of JPG images.

Figure 20 shows the quality values associated with the different formats for storage image sets for the optimal lighting and texture configurations previously examined in this study.

In comparison to JPG, the variation in the RMSE values for each specimen of the varied materials analyzed showed a slight improvement when TIFF was used. Such a trend is in line with the findings reported by Detchev *et al.* (2014), who observed that improvements in positional accuracy when using raw storage formats, as opposed to JPG, are typically of the order of submillimeter. Therefore, although TIFF images may yield marginal gains in positional accuracy, these gains are generally small when compared to the increased storage demand.

**Figure 21.**
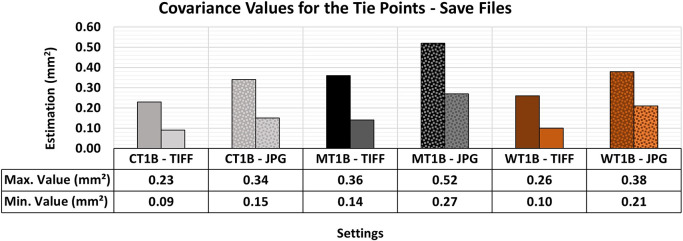
The maximum and minimum values obtained from the Variance and Covariance Matrix indicate the quality of adjustment across all materials when artificial texture T1 and lighting condition B (Vertical) were used. The TIFF configuration yielded better maximum and minimum values in the assessment compared to the respective 3D model that used JPG images.

The RMSE values did not reveal any significant differences that would justify the selection of a specific storage format. However, the results of the variance and covariance matrix showed substantial disparities, as shown in Figure 21. The 3D modeling with images in TIFF format showed a better adjustment quality compared to models using JPG images. This indicates that the primary advantage of TIFF lies in the internal consistency and robustness of the bundle adjustment rather than in improvements to absolute positional accuracy.

The maximum and minimum values of the variance and covariance matrix were notably more accurate and precise when the raw format was used. Morgan *et al.* (2017) reported this behavior, recognizing the higher level of detail in images in TIFF format and choosing raw formats over compressed ones. This decision was based on the superior capacity of TIFF for accommodating post-processing techniques while maintaining the integrity of the raw image data with no compression and information loss typical of JPG format.

TIFF storage format is recommended when post-processing flexibility and the stability of the adjustment are critical, especially in 3D modeling tasks requiring submillimeter precision. However, due to its rapid acquisition and lower storage space requirements, JPG can be a viable alternative in scenarios where extremely high precision is not mandatory or when storage and data transfer constraints are a priority.

### Limitations of the study

This study, centered on the three-dimensional modeling of objects within the context of laboratory structural testing, has identified some limitations inherent to the experimental approach, particularly with SfM techniques.

Capture Distance: The experimental protocol required photographic captures to be conducted at approximately 1 meter from the objects under study. This distance, dictated by stringent safety protocols in the laboratory environment, inevitably constrained the resolution and detail of the 3D models produced. It is recognized that shorter capture distances would likely enhance model quality by increasing image resolution and enabling a more comprehensive representation of the object. Consequently, it is imperative to establish minimum capture distances that meet safety standards and optimize the quality of 3D reconstructions.

Artificial Texture Patterns: The application of artificial texture patterns in this study was intended to enhance the visibility of surface features on the test specimens, a critical factor for SfM algorithms. However, closed or overly repetitive texture patterns can obscure finer surface details, such as cracks or microfractures, which are essential for accurate structural analysis. It is therefore crucial for laboratory professionals to select texture patterns that balance enhancing surface visibility and preserving the detectability of critical surface features. This selection is vital for ensuring the robustness of the 3D models generated through computer vision techniques. In this context, the proposed patterns should be understood as supportive tools rather than universal solutions, and their applicability depends on the inspection objective.

Auxiliary Lighting Positioning: The complex environment of the structural laboratory, characterized by the presence of various equipment and sensors, poses significant challenges for the effective positioning of auxiliary lighting. Inadequate lighting arrangements can introduce shadows and uneven illumination, which can degrade the quality of the images captured and, consequently, the accuracy of the 3D models produced. Proper lighting placement is essential to mitigate these effects, ensuring consistent illumination and minimizing shadowing that could compromise the integrity of the SfM process.

In summary, professionals engaged in such experimental work must possess a thorough understanding of the specific requirements and limitations of the tests being conducted, as well as the characteristics of the laboratory environment. This understanding is crucial to avoid suboptimal capture processes that could lead to reduced modeling quality or necessitate the repetition of experiments. By addressing these limitations, the fidelity and reliability of 3D models generated through SfM can be significantly improved.

## Conclusion

This study explored the impact of different configurations on the optimization of the close-range photographic capture process in indoor environments. The configurations were examined for the generation of high-quality image sets suitable for SfM technique in the 3D modeling of specimens and submillimeter positional accuracy required for laboratory structural testing.

To assess the quality levels achieved and identify the configurations that have the greatest impact on the photographic capture process, multiple capture sets were created using different lighting configurations, artificial textures patterns, and image storage formats for three varied specimens’ materials.

An analysis of the quality values suggested that more accurate results are obtained when Vertical and Adjacent auxiliary lighting models are used, since their adoption significantly improved the positional RMSE values and the oeverall quality of the model adjustment quality, especially for metallic specimens characterized by more uniform textures. However, specimens made of materials with high texture variation (e.g., concrete and wood) only showed significant improvements in the adjustment quality.

Artificial textures, characterized by checkered patterns with contrasting colors, applied to the surface of the specimens showed a behavior like that of auxiliary lighting. The benefits were associated with improvements in the quality of adjustment of the three-dimensional products generated, rather than with substantial changes in absolute positional accuracy. The combination of auxiliary lighting and artificial textures led to an approximately 40% improvement in modeling quality for materials with high texture variation. Conversely, for materials with a more uniform texture, such as the metallic sample, improvements in modeling quality reached around 60% when the two analyzed configurations were adopted.

The quality values obtained from the evaluation of two different image file formats, RAW (stored in TIFF) and lossless JPEG, indicate a slight superiority in the quality of 3D products for the RAW format (stored in TIFF) compared to the lossless JPEG file format. However, in situations where submillimeter accuracy is not required, the lossless JPEG format may be justified due to its smaller file size. Additionally, if lossy compression is used, it is recommended that the reader conducts a preliminary assessment of the quality of the results by employing the procedures and methods proposed in this research.

Overall, the analyses highlighted the improvements in the quality of 3D products obtained by SfM through the combined use of auxiliary lighting and artificial texture patterns. Regarding the storage format (TIFF or JPG), the results showed a slight advantage for TIFF in terms of model adjustment quality. However, it is the user’s responsibility to determine their specific requirements and assess whether the increased storage demand is justified, particularly when balancing precision needs against data storage and processing constraints.

As future work, ongoing efforts include the acquisition and evaluation of additional sensing technologies, such as stereo camera systems, dedicated optical configurations, and advanced illumination setups. In parallel, LiDAR-based depth sensing available in modern smartphones will be investigated as a complementary approach for selected laboratory-scale applications, enabling a broader comparison between passive and active 3D reconstruction techniques under structural testing conditions.

## Data Availability

OSFHome: Dataset of images SfM - FRM - Lighting and Artificial texture - JPG (DOI 10.17605/OSF.IO/K82AR) ( de Moraes 2024). The project contains the following underlying data:
• concrete test specimen (12 sets of images in JPG format, which varied according to the lighting and artificial texture configurations adopted in the study). • metal test specimen (12 sets of images in JPG format, which varied according to the lighting and artificial texture configurations adopted in the study). • wood test specimen (12 sets of images in JPG format, which varied according to the lighting and artificial texture configurations adopted in the study). Data are available under the terms of the Creative Commons Attribution 4.0 International license (CC-BY 4.0). Zenodo: Optimizing Submillimeter 3D Modeling with Auxiliary Lighting and Artificial Textures: An SfM-Based Approach Creators, DOI: https://doi.org/10.5281/zenodo.13937284 The project contains the following reporting guidelines:
• STROBE Statement Utilized in the Preparation of the Article Titled “Optimizing Submillimeter 3D Modeling with Auxiliary Lighting and Artificial Textures: An SfM Approach” Data are available under the terms of the Creative Commons Attribution 4.0 International license (CC-BY 4.0).
