# Supplementary material for: Optimizing Submillimeter 3D Modeling with Auxiliary Lighting and Artificial Textures: An SfM-Based Approach
Source: F1000Res. 2026 Feb 10;13:1479. Originally published 2024 Dec 4. [Version 2] doi: 10.12688/f1000research.157676.2 (PMC13022561; doi:10.12688/f1000research.157676.2)
Supplement: Peer review history

# Reviewer report: Reviewer response for version 2

**DOI:** 10.5256/f1000research.195215.r468199

**Published:** 2026-03-26

**Authors:** Abdurahman Yasin YİĞİT

**Affiliations:** 1 Mersin University, Mersin, Turkey

The manuscript has a meaningful practical contribution, but the wording still sometimes overreaches the actual validation framework. The study mainly evaluates distance-based RMSE from control bars and bundle-adjustment/internal precision indicators derived from the variance–covariance matrix, not full-surface geometric fidelity against an external reference model. Since the authors themselves acknowledge that no full geometric ground truth was available, the wording around “submillimeter 3D model accuracy” and “3D model quality” should be slightly softened and framed more precisely as improvement in distance-based scaling accuracy and adjustment robustness under the tested conditions.

 The manuscript repeatedly states that the combination of auxiliary lighting and artificial textures improved modeling quality by approximately 40% for high-texture materials and 60% for the more uniform metal surface. This is potentially important, but I could not find a sufficiently explicit definition of how these percentages were calculated, what baseline was used, and which specific metric represents “modeling quality” in this percentage statement. Please define this clearly in the Methods or Results and ensure the same metric is used consistently in the Abstract and Conclusion.

 The terminology used for the file-format comparison should be corrected. According to the Methods, the photographs were originally acquired in RAW and then converted to JPG and TIFF for comparison. Therefore, expressions such as “RAW (stored in TIFF)” are technically confusing. TIFF is not the native RAW capture format; it is an exported/converted working format. This distinction is important, because the paper is making a methodological claim about file formats. In the same vein, the manuscript also refers to “lossless JPEG,” which requires clarification because standard JPEG is normally lossy unless a very specific workflow is being used.

 Because the TIFF vs JPG comparison is one of the paper’s core claims, the image-conversion workflow needs more detail. Please report the exact export settings used in Photoshop: TIFF bit depth, JPG quality/compression level, color space, white balance handling, sharpening, noise reduction, and any demosaicing or tonal adjustments applied during conversion. Without these details, the file-format comparison remains only partially reproducible.

 Reproducibility is still incomplete for the file-format experiment. The data availability section explicitly lists JPG image sets as underlying data, but the manuscript also draws conclusions from the TIFF comparison. If TIFF exports or the original RAW files are not shared, then the TIFF/JPG experiment cannot be fully reproduced by other researchers. This should be corrected either by sharing the TIFF and/or RAW material, or by explicitly acknowledging that the file-format comparison is only partially reproducible in the current repository state.

 The use of the maximum and minimum diagonal values of the variance–covariance matrix as indicators of adjustment quality needs a more rigorous methodological explanation. At present, the paper states that these values are used to describe adjustment stability/internal precision, but it is still unclear from which exact Metashape output they were extracted, whether they refer to tie-point-related uncertainty or another matrix, and why the maximum/minimum diagonal values were selected instead of more conventional aggregate descriptors. Please explain this more explicitly so that readers can understand and reproduce the metric.

 There is a notable inconsistency in the coding of lighting configurations. Table 1 lists Standard (A), Vertical (B), Adjacent (D), Beneath (E), while Figure 3 visually presents the configurations as panels (a) to (d), and later the discussion refers to Vertical (B) and Adjacent (C) as the preferred configurations. This inconsistency should be fully harmonized across the table, figure, captions, and discussion text. At the moment, it is unnecessarily confusing for the reader.

 Some figure captions still contain obvious carry-over errors. For example, on the page showing the metal lighting assessment, the caption of Figure 9 still refers to a “concrete sample,” and Figure 10 mentions the “concrete object” while the plotted material is metal. These caption mistakes reduce confidence in the precision of the presentation and should be carefully corrected throughout the manuscript.

 The interpretation of the sparse-cloud comparison should be made more cautious. Absolute sparse-point counts are influenced not only by lighting configuration but also by the inherent texture richness of the material itself. Therefore, Figure 13 is useful descriptively, but it should not be treated as a standalone proof of lighting superiority unless this is better normalized or more carefully qualified in the text.

 The explanation for using a regular grid capture without camera-axis rotation has been addressed in the author response, but the main manuscript would still benefit from a short self-contained explanation. Since this capture geometry can be questioned by close-range SfM readers, a concise justification should appear directly in the Methods, rather than depending mainly on earlier work by the same authors.

 In several places, the discussion should emphasize that the strongest gains are often observed in adjustment robustness/internal precision, whereas the RMSE changes are sometimes small, especially for concrete and wood. This distinction is already partly recognized in the revised manuscript, but it should be carried more consistently into the wording of the Abstract, Results, and Conclusion so that readers do not interpret all improvements as equally strong gains in absolute geometric accuracy.

 The manuscript is now close to being indexable, but it would become much stronger with one final editorial sweep focused on consistency: terminology, figure captions, configuration coding, and the exact definition of each evaluation metric. The technical content is useful, but the presentation still needs tightening to match the improved methodological transparency of the revised version.

Is the work clearly and accurately presented and does it cite the current literature?

Yes

If applicable, is the statistical analysis and its interpretation appropriate?

No

Are all the source data underlying the results available to ensure full reproducibility?

No

Is the study design appropriate and is the work technically sound?

Yes

Are the conclusions drawn adequately supported by the results?

Partly

Are sufficient details of methods and analysis provided to allow replication by others?

No

Reviewer Expertise:

GIS and Remote sensing

I confirm that I have read this submission and believe that I have an appropriate level of expertise to confirm that it is of an acceptable scientific standard, however I have significant reservations, as outlined above.

# Reviewer report: Reviewer response for version 1

**DOI:** 10.5256/f1000research.173163.r402790

**Published:** 2025-08-23

**Authors:** Ivan Nikolov

**Affiliations:** 1 Aalborg University, Aalborg, Denmark

The paper looks into how lighting placement and surface texture change the quality of close range SfM 3D reconstructions on three types of materials - wood, concrete, metal. The research is quite interesting and can be very useful as an overview of the requirements that engineers should have to do with their setups before they can extract good 3D surface analysis captures.

 There are some parts in the paper that are currently lacking and require additional work:

 The authors briefly discuss their choice of of Agisoft Metashape Pro. Even though it's extensively cited in the literature the software is extremely expensive (especially the pro version) and it has comparative results to other free software solutions like Meshroom, RealityScan, 3DS Zephyr that are free. It also has comparable results to Pix4D, another paid software solution. The authors should either compare the results to at least the free SFM software out there or cite research that compares it. The reviewer has proposed paper citations for the latter option (please check). 

 In addition, no explanation has been given on the specific settings used in Metashape Pro to obtain the reconstructions, making the replicability impossible.

 Furthermore, the authors should discuss why not use other ways for 3D reconstruction for indoor close range, like structured light, stereo cameras, time-of-flight cameras, solid state lidars, etc.

 For the testing, the authors need to mention the light luminosity used in their research again to have better replicability.

 It is generally not advised to take photos of objects for SfM reconstruction by just taking them in a line with overlap, without any rotation in the camera. This most of the time results in poorer reconstruction than if you also have a rotation in the camera axis. Why did the authors choose to do this capture configuration?

 Do the authors also have ground truth representations of the objects that are being scanned? Maybe captured through a different scanning method or having 3D CAD models. Normally, to better compare 3D reconstruction quality, a comparison to ground truth objects is advised. Then the distance between the objects can be calculated, there can be a comparison where the errors are on the surface of the object, how much noise there is, etc. It will be a good idea to have such a comparison in the paper.

 Authors are using a caliper to capture real-life distances. How many measurements were done for each place of measurement? Normally, when a human-led measurement like this is done, we need to know the standard deviation of the human error. These errors have been shown to propagate through the calculations and make the scaling of 3D model have errors (please check the third proposed article for more information)

 When applying the texture to the surfaces, the reviewer imagines it was done by hand. This can lead to errors and places in the 3D reconstruction that have noise or holes. Why wasn't something like a projector or laser projector used to project the patterns on the surface? Then the uniformity of the pattern would be guaranteed. 

 Currently, there are no visuals of the reconstructions from the different experiments, making it hard to visually judge how well they are achieved. Please add these.

Is the work clearly and accurately presented and does it cite the current literature?

Partly

If applicable, is the statistical analysis and its interpretation appropriate?

Not applicable

Are all the source data underlying the results available to ensure full reproducibility?

No

Is the study design appropriate and is the work technically sound?

Yes

Are the conclusions drawn adequately supported by the results?

Yes

Are sufficient details of methods and analysis provided to allow replication by others?

Partly

Reviewer Expertise:

Deep learning, 3D reconstruction, SfM, photogrammetry, computer graphics

I confirm that I have read this submission and believe that I have an appropriate level of expertise to confirm that it is of an acceptable scientific standard, however I have significant reservations, as outlined above.

## References

[1] : Comparative analysis of digital photogrammetry software for cultural heritage. *Digital Applications in Archaeology and Cultural Heritage* .2020;18: 10.1016/j.daach.2020.e00157 10.1016/j.daach.2020.e00157

[2] : Benchmarking Close-range Structure from Motion 3D Reconstruction Software Under Varying Capturing Conditions.10058: 10.1007/978-3-319-48496-9_2 15-26 10.1007/978-3-319-48496-9_2

[3] : Calculating Absolute Scale and Scale Uncertainty for SfM Using Distance Sensor Measurements. 10.4018/978-1-5225-5294-9.ch008 168-192 10.4018/978-1-5225-5294-9.ch008

# Response

**Published:** 2026-01-12

**Authors:** Francisco Roza de Moraes

**Affiliations:** Transportation Engineering, Universidade de Sao Paulo Escola de Engenharia de Sao Carlos, São Carlos, State of São Paulo, Brazil

We thank the reviewer for the detailed and technically insightful comments, which significantly improved the methodological transparency and robustness of the manuscript. Responses to each comment are provided below.

**Comment 1**

*The authors briefly discuss their choice of Agisoft Metashape Pro. Even though it is extensively cited in the literature, the software is extremely expensive (especially the Pro version) and has comparable results to free SfM solutions such as Meshroom, RealityScan, and 3DF Zephyr, as well as to other commercial software such as Pix4D. The authors should either compare the results with at least free SfM software or cite research that performs such comparisons.*

**Response:**

 We thank the reviewer for this important and constructive comment. The suggested references comparing Agisoft Metashape with alternative SfM solutions were carefully reviewed and incorporated into the revised manuscript.

 The choice of Agisoft Metashape Pro in this study was primarily motivated by its availability in our laboratory infrastructure and by the research team’s prior experience with the software, which facilitated a consistent and controlled experimental workflow. Importantly, the objective of the study was not to benchmark SfM software packages, but rather to investigate the influence of photographic acquisition conditions, such as auxiliary lighting, artificial texture patterns, and storage formats, on the quality of close-range SfM-based 3D reconstructions.

 As clarified in the revised manuscript, other computational solutions, both commercial and open source (e.g., Meshroom, COLMAP, OpenMVG, RealityScan, and 3DF Zephyr), can produce comparable results and would also meet the methodological requirements of the proposed experiments. The conclusions drawn in this work are therefore not software-dependent and can be transferred to alternative SfM pipelines.

**Comment 2**

*In addition, no explanation has been given on the specific settings used in Metashape Pro to obtain the reconstructions, making the replicability impossible.*

**Response:**

 We thank the reviewer for highlighting this important point regarding replicability. In the revised version of the manuscript, the processing workflow and the specific configuration parameters used in Agisoft Metashape Pro were explicitly described.

 A dedicated paragraph was added to the **Methods** section detailing the alignment, dense cloud generation, mesh reconstruction, and tiled model parameters applied consistently across all datasets. These settings were kept fixed for all experiments to ensure comparability between lighting configurations, artificial texture patterns, and storage formats.

 By explicitly reporting these parameters, the revised manuscript now allows full replication of the reconstruction workflow using Agisoft Metashape Pro or equivalent SfM software.

**Comment 3**

*Furthermore, the authors should discuss why not use other ways for 3D reconstruction for indoor close range, like structured light, stereo cameras, time-of-flight cameras, solid-state lidars, etc.*

**Response:**

 We thank the reviewer for this relevant and insightful comment. The choice of Structure-from-Motion (SfM) as the core reconstruction technique in this study was deliberate and aligned with the broader objectives of the doctoral research from which this work originates.

 The primary aim of this research was to investigate how photographic acquisition conditions, such as auxiliary lighting, artificial texture patterns, and image storage formats, affect the quality and reliability of SfM-based 3D reconstructions in laboratory-scale structural testing environments. SfM was selected because it is a passive, image-based technique that combines flexibility, scalability, and relatively low hardware cost, while allowing the use of standard photographic equipment and adaptable camera positioning under laboratory safety constraints.

 Alternative close-range 3D reconstruction approaches, including structured light systems, stereo camera setups, time-of-flight sensors, and solid-state LiDARs, were considered within the broader research framework and, in some cases, evaluated in separate experimental stages. However, these techniques typically require specialized and often costly hardware, controlled illumination conditions, fixed sensor geometries, or limited operational ranges, which can reduce their applicability in structural laboratories characterized by space limitations, safety restrictions, and variable experimental configurations.

 As future work, the research group is currently acquiring additional equipment, including stereo camera systems, dedicated lenses, and advanced illumination setups, which will allow a systematic comparison between SfM and alternative close-range 3D sensing technologies. Additionally, LiDAR-based depth sensing, available in modern smartphones, will be investigated as a complementary solution for selected laboratory scenarios. These investigations will be reported in future publications.

 To clarify the scope of the present study, a brief discussion was added to the revised manuscript emphasizing that the objective was not to benchmark different 3D reconstruction technologies, but to optimize SfM acquisition strategies within realistic laboratory constraints.

***Comment 4***

*For the testing, the authors should reiterate the light luminosity used in their research to enhance replicability.*

***Response:***

 We thank the reviewer for this comment regarding replicability. In the revised version of the manuscript, the auxiliary lighting specifications were explicitly reported.

 The manuscript now states that two auxiliary lighting units (softboxes) were used, each equipped with a 7,000-lumen LED lamp and a color temperature of 5,000 K. These parameters were kept constant across all experiments involving auxiliary lighting to ensure consistency and reproducibility.

 By explicitly reporting both the luminous flux and the color temperature of the light sources, the revised manuscript provides sufficient information for other researchers to replicate the illumination conditions adopted in this study.

**Comment 5**

*It is generally not advised to take photos of objects for SfM reconstruction by just taking them in a line with overlap, without any rotation in the camera. This most of the time results in poorer reconstruction than if you also have a rotation in the camera axis. Why did the authors choose to do this capture configuration?*

**Response:**

 We thank the reviewer for this important methodological observation. The capture configuration adopted in this study was not arbitrary but was based on previous experimental evaluations conducted by the authors.

 The use of a controlled capture geometry with overlapping images acquired along a regular grid, without intentional camera axis rotation, was motivated by earlier investigations reported by Moraes and da Silva, in which different acquisition strategies were systematically tested under similar laboratory conditions. In those experiments, no significant improvements in positional accuracy or model adjustment quality were observed when random camera orientations or additional rotations were introduced.

 Given that the primary objective of the present study was to isolate and evaluate the effects of auxiliary lighting, artificial texture patterns, and image storage formats on SfM reconstruction quality, a controlled and repeatable acquisition strategy was adopted. This approach reduced additional sources of variability and facilitated direct comparison between experimental configurations.

 In addition, the selected capture strategy reflects practical constraints commonly encountered in laboratory structural testing, where camera positioning is often limited by safety protocols, equipment layout, and restricted access around the specimen. Under these conditions, a regular and well-controlled acquisition geometry provides a robust and reproducible solution without compromising the validity of the comparative analyses performed.

**Comment 6**

*Do the authors also have ground truth representations of the objects that are being scanned? Maybe captured through a different scanning method or having 3D CAD models. Normally, to better compare 3D reconstruction quality, a comparison to ground truth objects is advised. Then the distance between the objects can be calculated, and there can be a comparison where the errors are on the surface of the object, how much noise there is, etc. It will be a good idea to have such a comparison in the paper.*

**Response:**

 We thank the reviewer for this relevant and constructive suggestion. In the present study, a full geometric ground truth representation of the specimens, such as high-resolution 3D CAD models or reference scans obtained with alternative sensing technologies, was not available.

 Instead, the assessment of reconstruction quality was based on physically measurable reference elements incorporated into the experimental setup. Scale bars and control bars with known lengths were positioned within the region of interest and measured using a high-precision digital caliper. These elements provided reliable reference values for evaluating positional accuracy through distance-based metrics, such as RMSE, which are commonly adopted in close-range photogrammetry and SfM-based studies.

 This approach was selected because the primary objective of the research was not to perform a full surface-to-surface comparison between reconstructed models and an external ground truth, but rather to analyze the relative impact of different acquisition conditions, including lighting configurations, artificial texture patterns, and storage formats, under controlled and repeatable laboratory conditions.

 While surface-based comparisons against a full geometric ground truth can provide additional insights into noise distribution and local deviations, implementing such analyses would require complementary sensing systems or reference models that were outside the scope of the present work. This limitation is now explicitly acknowledged in the manuscript.

**Comment 7**

*Authors are using a caliper to capture real-life distances. How many measurements were done for each place of measurement? Normally, when a human-led measurement like this is done, we need to know the standard deviation of the human error. These errors have been shown to propagate through the calculations and cause errors in the scaling of the 3D model have errors.*

**Response:**

 We thank the reviewer for this important comment regarding measurement uncertainty and error propagation.

 In the present study, each scale bar and control bar was measured **five independent times** using a high-precision digital caliper. The repeated measurements allowed the estimation of the mean value and the corresponding standard deviation for each reference length. The observed standard deviations were on the order of **hundredths of a millimeter**, which is consistent with the manufacturer’s specifications of the instrument and within the accuracy level required for the submillimeter analyses performed in this work.

 These reference measurements were subsequently used for model scaling and accuracy assessment. By relying on averaged values obtained from repeated measurements, the influence of operator-induced variability was minimized. The resulting uncertainty associated with the physical measurements was therefore significantly smaller than the variations observed in the SfM reconstruction metrics, ensuring that the scaling process did not dominate the error budget of the 3D models.

 This measurement strategy is consistent with common practices in close-range photogrammetry and laboratory-scale SfM studies, where repeated caliper measurements are used to control human-induced uncertainty and ensure reliable reference data for model evaluation.

**Comment 8**

*When applying the texture to the surfaces, the reviewer imagines it was done by hand. This can lead to errors and places in the 3D reconstruction that have noise or holes. Why wasn't something like a projector or a laser projector used to project the patterns on the surface? Then the uniformity of the pattern would be guaranteed.*

**Response:**

 We thank the reviewer for this relevant observation. The use of projected patterns, including digital or laser projectors, was considered during the experimental design phase of this research.

 However, the adoption of projected textures was not pursued due to practical and methodological constraints associated with the laboratory environment and the objectives of the study. First, the use of projectors directly conflicted with the auxiliary lighting configurations under investigation. Since this work explicitly evaluates the influence of different lighting arrangements on SfM reconstruction quality, introducing a projected pattern would act as an additional and uncontrolled light source, altering the illumination distribution, contrast, and radiometric consistency of the scene. This would compromise the isolation of lighting-related variables that were central to the experimental design.

 Second, the experimental protocol required a relatively large number of image acquisitions for each configuration, all performed using a single camera and under strict safety and access constraints typical of structural testing laboratories. During preliminary tests, maintaining consistent projection geometry and intensity over extended acquisition times proved challenging. Small changes in projector alignment, occlusions caused by camera repositioning, or gradual variations in projection intensity over time introduced inconsistencies that negatively affected feature detection and image matching.

 In addition, the presence of reference elements such as scale bars and control bars with checkerboard patterns posed further challenges. Projected patterns interfered with the automatic detection of these reference targets, reducing their reliability for scaling and accuracy assessment.

 For these reasons, manually applied artificial texture patterns were selected as a controlled and stable alternative. Although hand-applied patterns may introduce local variability, they provided consistent contrast throughout the acquisition process, did not interfere with the auxiliary lighting configurations, and ensured reliable detection of reference elements across all datasets.

 To clarify this methodological choice, the revised manuscript explicitly defines artificial textures as manually applied patterns and acknowledges the trade-offs associated with alternative projection-based approaches.

# Reviewer report: Reviewer response for version 1

**DOI:** 10.5256/f1000research.173163.r356962

**Published:** 2025-02-10

**Authors:** Aleksandar Ašonja

**Affiliations:** 1 University Business Academy, Cvećarska, Serbia

In general, the research topic is very current. Some chapters of the manuscript need to be revised to make them clearer and generally acceptable to readers. I suggest the following minimal changes and additions to make the manuscript acceptable for indexing. 

 1) Do not write the manuscript in personal pronouns (we…..) and possessive adjectives (Our…… ). The manuscript should be written in the third person and in the past tense.

 2) The title and keywords describe the research well.

 3) The proposal is to expand the abstract with some of the research results.

 4) The introductory chapter is clearly written. This chapter should be expanded by introducing more recent research.

 5) The work methodology is not very clear. In the work methodology, it should be noted: What scientific methods, techniques, analyses, software, devices and other equipment are used for research.

 6) The research results are clear and describe the research well.

 7) In the conclusion, it should be stated what was the scientific justification of the research.

 8) The conclusion should highlight what the continuation of the research could be.

 9) The reference should be expanded by introducing additional references.

Is the work clearly and accurately presented and does it cite the current literature?

Yes

If applicable, is the statistical analysis and its interpretation appropriate?

Yes

Are all the source data underlying the results available to ensure full reproducibility?

Yes

Is the study design appropriate and is the work technically sound?

Yes

Are the conclusions drawn adequately supported by the results?

Yes

Are sufficient details of methods and analysis provided to allow replication by others?

Yes

Reviewer Expertise:

Industrial Engineering, Mechanical Engineering, Renewable Energy Sources, Agricultural Engineering,Energy and Environment.

I confirm that I have read this submission and believe that I have an appropriate level of expertise to confirm that it is of an acceptable scientific standard.

# Response

**Published:** 2026-01-12

**Authors:** Francisco Roza de Moraes

**Affiliations:** Transportation Engineering, Universidade de Sao Paulo Escola de Engenharia de Sao Carlos, São Carlos, State of São Paulo, Brazil

We thank the reviewer for the careful evaluation of the manuscript and for the constructive comments, which helped improve clarity, structure, and scientific rigor. Each comment is addressed point by point below.

**Comment 1**

*Do not write the manuscript in personal pronouns (we…). The manuscript should be written in the third person and in the past tense.*

**Response:**

 The manuscript was revised to remove personal pronouns and to ensure consistent use of the third person and past tense throughout all sections, following standard scientific writing conventions.

**Comment 2**

*The proposal is to expand the abstract with some of the research results.*

**Response:**

 The abstract was expanded to include a concise summary of the main experimental results, highlighting the effects of auxiliary lighting, artificial texture patterns, and image storage formats on SfM-based 3D modeling quality.

**Comment 3**

*The introductory chapter is clearly written. This chapter should be expanded by introducing more recent research.*

**Response:**

 The Introduction was expanded with additional recent and relevant references addressing close-range SfM applications, lighting conditions, and feature detection robustness, strengthening the contextual background of the study.

**Comment 4**

*The work methodology is not very clear. In the work methodology, it should be noted: What scientific methods, techniques, analyses, software, devices, and other equipment are used for research.*

**Response:**

 The Methods section was revised to improve clarity and reproducibility. The experimental setup, lighting configurations, artificial texture patterns, camera parameters, capture distance, calibration strategy, and evaluation metrics were explicitly described.

**Comment 5**

*It should be specified which materials, software, devices, and other equipment are used for research.*

**Response:**

 All materials, software, and equipment used in the study were explicitly detailed, including camera and lens specifications, auxiliary lighting characteristics, calibration instruments, and processing software.

**Comment 6**

*In the conclusion, it should be stated what the scientific justification of the research.*

**Response:**

 The Conclusion was revised to clearly state the scientific contribution of the study, emphasizing the systematic evaluation of capture configurations for improving SfM-based 3D modeling accuracy in laboratory environments.

**Comment 7**

*The conclusion should highlight what the continuation of the research could be.*

**Response:**

 The Conclusion now includes perspectives for future research, such as extending the analysis to other materials, capture distances, texture strategies, and laboratory conditions.

**Comment 8**

*The reference should be expanded by introducing additional references.*

**Response:**

 The reference list was expanded with additional recent and relevant publications to better support the discussion and contextualize the findings.
